# Host Cavities Enhance the Photocatalytic Conversion of *α*‐Terpinene to Ascaridole Under Visible‐Light Irradiation

**DOI:** 10.1002/chem.202502113

**Published:** 2025-09-02

**Authors:** Mawgan U. Main, Dominic Taylor, Leonardo Amicosante, John M. Tobin, Callum M. S. Jones, Jose Marques‐Hueso, Laura J. McCormick McPherson, Simon J. Teat, David Ellis, Martin J. Paterson, Filipe Vilela, Scott J. Dalgarno

**Affiliations:** ^1^ Institute of Chemical Sciences Heriot‐Watt University Riccarton Edinburgh EH14 4AS UK; ^2^ Current address: EastCHEM School of Chemistry The University of Edinburgh David Brewster Road Edinburgh Scotland EH9 3FJ UK; ^3^ Institute of Sensors Signals and Systems Heriot‐Watt University Riccarton Edinburgh EH14 4AS UK; ^4^ Current address: Clare College University of Cambridge Trinity Lane Cambridge UK; ^5^ Current address: Institute of Material Science of the University of Valencia C/ Catedrático José Beltrán Martinez, 2 Paterna Valencia 46980 Spain; ^6^ Lawrence Berkeley National Laboratory 1 Cyclotron Road, MS 6R2100 California 94720 USA

**Keywords:** BODIPY, calix[4]arenes, photocatalysis, singlet oxygen

## Abstract

BODIPY‐functionalized host molecules have been used as effective visible‐light photosensitizers in the conversion of α‐terpinene to ascaridole in the presence of molecular oxygen. Host‐guest interactions enhance the effective local concentration of the substrate and singlet oxygen generated by the photosensitizing host. This results in up to a 28‐fold increase in the rate of conversion depending on the host employed. A tetramethyl BODIPY analogue, in which guest access to the host cavity is blocked, was also employed to confirm that the cavities do indeed enhance substrate turnover. Whilst the latter photocatalyst is more efficient due to the suppression of nonradiative relaxation pathways associated with rotation of the *meso*‐phenyl substituent, the rate enhancement induced by host‐guest binding is not present. Combined, this provides insight for the future design of enhanced systems.

## Graphical abstract

BODIPY‐functionalized host molecules are effective visible‐light photosensitizers for the conversion of *α*‐terpinene to ascaridole in the presence of molecular oxygen, providing up to a 28‐fold increase in the rate of conversion depending on the host employed.

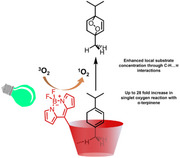

## 1 Introduction

Supramolecular catalysis is a field that continues to attract significant attention, reasons for which include the fact that weak interactions, molecular encapsulation, and complex systems deliver notable results when applied to appropriate synthetic transformations.^[^ ^1^ ^]^ Product inhibition can be detrimental in such systems and can occur due to, for example, strong host‐guest complex formation. Examples of strategies used to circumvent this problem include targeting weak host‐guest interactions in system design, engaging the product in an onward reaction external to the host, or performing a reaction in which the product binds less well than the reactants.

With the thrust of this contribution in mind (vide infra), it is worth mentioning literature examples concerned with the concept of raising the effective local concentration of reactants in bimolecular reactions. An excellent early example relating to encapsulation is the acceleration of a Diels‐Alder reaction within a self‐assembled capsule as reported by Rebek.^[^ ^2^ ^]^ Although product inhibition was observed, the system displayed two orders of magnitude enhancement due to encapsulation. Another excellent example is the report by Fujita and co‐workers in which they use self‐assembled cages or bowls to achieve either unusual regioselectivity in or efficient catalysis of a Diels‐Alder reaction.^[^ ^3^ ^]^ Notably, the ligands employed in cage/bowl assembly are polyaromatic in nature, presenting hydrophobic pockets in these assemblies that can be exploited for fascinating host‐guest chemistry. In the case of the bowl (present at 10 mol%), this was found to operate through enhanced concentration to effectively catalyze the reaction of a wide range of anthracene and phthalimide derivatives, with turnover in this case being attributable to weak host‐guest (H:G) binding due to the products being bent and no longer able to form *π*‐stacking interactions. Enhancement of reactivity due to H:G interactions can also be exploited within photochemical processes, whereby guests within the confines of their hosts (mainly within cyclodextrins, cucurbiturils, and Pd nanocages) can undergo chemical transformations upon visible light irradiation.^[^ ^4^ ^]^ However, these reactions mainly utilize high‐energy UV‐light and are noncatalytic in nature due to the strong interactions that drive H:G complex formation.^[^ ^5^ ^]^

**Figure 1:**
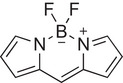
Chemical structure of the parent BODIPY core.

Moving away from exploiting the native photochemistry of the guest, we have been exploring the reaction of guest molecules with singlet oxygen (^1^O_2_). Excitation of ground triplet state molecular oxygen (^3^O_2_) to its electronically excited state increases its reactivity, and thus ^1^O_2_ has important applications in photodynamic therapy (PDT), fine chemical synthesis, water treatment etc.^[^ ^6^ ^]^ The simplest method to generate ^1^O_2_ is by using a photosensitizer, which can absorb light at a specific wavelength, exciting it to a higher energy state (^1^PS*). Intersystem crossing (ISC) leads to the occupation of the triplet state (^3^PS*) which in the presence of ^3^O_2_ can then undergo triplet‐triplet annihilation (TTA) to generate singlet oxygen and restore the photosensitizer to its ground state (^1^PS).^[^ ^7^ ^]^ One class of dyes that has been proven to be effective photosensitizers of oxygen is the family of dyes based on the 4,4‐difluoro‐4‐bora‐3*a*,4*a*‐diaza‐*s*‐indacene (BODIPY) core, which exhibit properties such as favorable absorption wavelengths (>450 nm), high molar attenuation coefficient, and photochemical stability (Figure 1).^[^ ^8^ ^]^

**Scheme 1:**
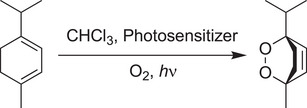
Synthesis of ascaridole from *α*‐terpinene via the photosensitization of singlet oxygen under visible light irradiation.

Herein we detail BODIPY‐functionalized calix[4]arene host molecules with a view to exploring the effects that cavities may exert over the well‐known bimolecular photocatalytic conversion of *α*‐terpinene to ascaridole (Scheme 1). This reaction is an excellent tool for monitoring the generation of ^1^O_2_ as conversion can be readily determined with the use of convenient ^1^H NMR handles. Our primary strategy in system design was to utilize host‐guest interactions between the substrate and the calix[4]arene cavity to a) increase the effective concentration of *α*‐terpinene and ^1^O_2_ generated at the upper rim of the host, thus enhancing photocatalysis, and b) potentially mitigate product inhibition by avoiding strong host‐guest complex formation with the product (which we anticipated would bind less favorably due to both a change in shape and knowledge of calix[4]arene host‐guest behaviors).

## 2 Results and Discussion

**Figure 2:**
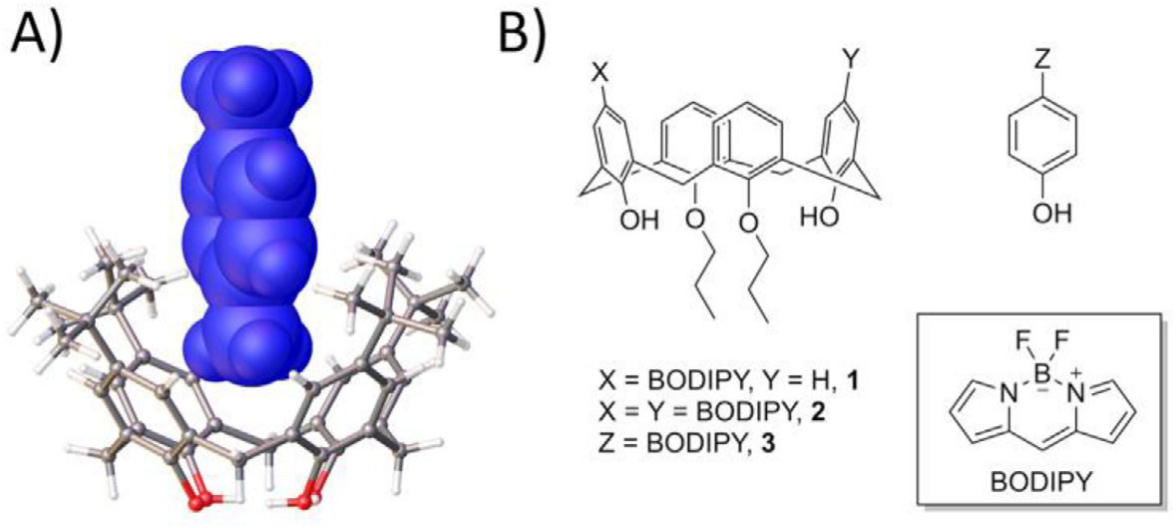
A) A host‐guest complex between TBC[4] and *p*‐xylene showing the guest in space‐filling representation.^[^ ^10^ ^]^ B) The three BODIPY‐containing target molecules selected for this study.

With respect to the targeted H:G chemistry, the structural similarity between *α*‐terpinene and toluene/*p*‐xylene makes it an ideal candidate for a study with calix[4]arene‐based photosensitizer hosts. For example, very early studies concerned with the host‐guest chemistry of *p*‐tert‐butylcalix[4]arene (TBC[4]) include a structural report on the toluene solvate^[^ ^9^ ^]^ and other H:G complexes formed with structurally related guests due to complementarity in both shape and size (e.g., the *p*‐xylene complex shown in Figure 2A).^[^ ^10^ ^]^ With all of the above in mind, we arrived at three initial target molecules, C[4]s appended at the upper‐rim with either one or two BODIPY moieties (**1** and **2**, respectively), as well as an equivalent photosensitizer lacking a cavity (**3**) that would act as a “blank” (Figure 2B). Distal 25,27‐*di*‐propoxy‐calix[4]arene (DiPrC[4]) was targeted as the primary host scaffold, as introduction of the alkyl chains at the lower‐rim improves solubility whilst simultaneously retaining a cavity for facile guest occupation; the cavity is stabilized by H‐bonding from the two remaining phenolic groups.

A literature search for C[4]s that are both in a cone conformation and appended at the upper rim with BODIPY moieties returned very few reports.^[^ ^11^, ^12^, ^13^, ^14^ ^]^ Inspection of these showed the main focus to be on their use as pH or metal ion binding sensors,^[^ ^12^, ^13^, ^14^ ^]^ but there has (to our knowledge) been no examination of their potential use as photosensitizing catalysts operating through H:G complexation. The results from our search did return the synthesis and structural characterization of a dichloromethane (DCM) solvate of **2**, but no subsequent use of this compound.^[^ ^11^ ^]^

### 2.1 Synthesis

**Figure 3:**
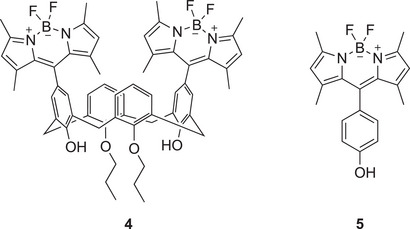
Tetramethyl‐BODIPY analogues, **4** and **5**, of compounds **2** and **3**, respectively (Figure 2B).

Compounds **1** – **3** were prepared from their corresponding formyl precursors^[^ ^15^, ^16^ ^]^ in line with literature precedent (Supporting Information Section 1), and their purity was checked by standard analytical techniques. The use of 2,4‐dimethylpyrrole in BODIPY synthesis has been known to afford improved yields relative to pyrrole. We initially avoided this in our target design due to the known occupation of the C[4] cavity by 4‐methyl groups of the BODIPY moieties,^[^ ^12^, ^13^, ^14^ ^]^ but were nevertheless interested in the behavior of compounds **4** and **5** (Figure 3) for useful comparison with that of **1** – **3**. In our hands the synthesis of **4** proved to be challenging, and we were only able to obtain a pure sample in very low yield. However, multiple syntheses afforded a sufficient amount of pure **4** to allow us to study its behavior as a photosensitizer in the reaction shown in Scheme 1.

### 2.2 Structural Studies

**Figure 4:**
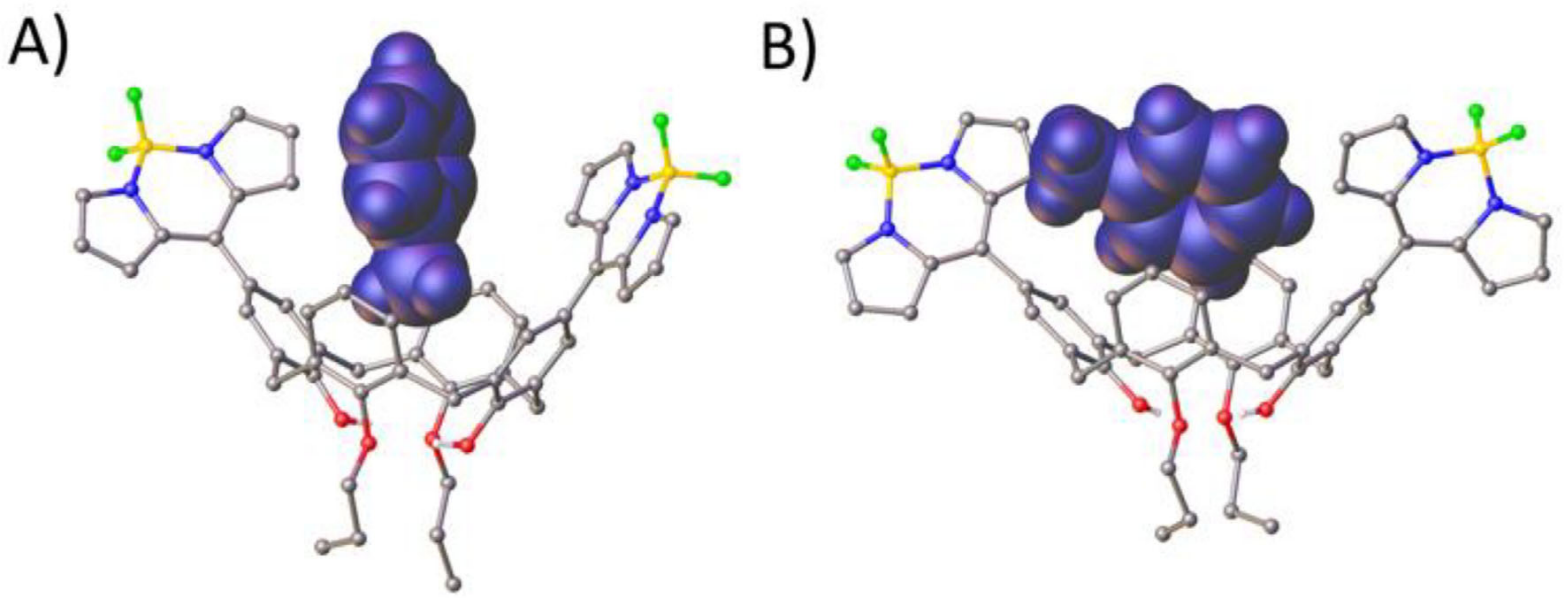
Single crystal X‐ray structures of **2** with toluene guest molecules oriented vertically A) or at an angle B) within the C[4] cavity. Toluene guests are shown in space‐filling representation. H atoms except those of the guest and lower‐rim hydroxyl groups are omitted for clarity.

Crystallization of **1** and **2** was attempted by vapor diffusion from a range of solvents with varied size and character (toluene, DCM, chloroform, or mesitylene), in all cases using petroleum ether as a counter solvent. Unfortunately, both **1** and **2** were insoluble in *α*‐terpinene so, precluded any direct observation of cavity occupation, and any attempts to include this in a crystallization mixture were unsuccessful. Single crystals suitable for diffraction studies were obtained for **1** from toluene or mesitylene and for **2** from DCM and toluene. All structures (see Supporting Information, Figures S1–S6) show that the cavities are present and stabilized by H‐bonding between the lower‐rim hydroxyl/distal propoxy groups (thus remaining accessible for guest occupation), and of particular interest is the crystallization of **2** from toluene. In this case two crystal morphologies were found upon inspection, and structural analysis revealed that the toluene is able to occupy the C[4] cavity in two different orientations, giving rise to two crystal forms as a result. Figure 4 shows the toluene guest molecules in space‐filling representation. In the vertical orientation (Figure 4A), the toluene guest shows a key CH… *π* interaction occurring from the methyl group to an aromatic ring of the host with a CH…centroid distance of 2.696 Å. The side‐on toluene complex (Figure 4B) shows two CH…*π* interactions occurring between aromatic hydrogens of the guest and aryl rings of the host with CH…centroid distances of 2.785 and 2.737 Å. The closest H:G *π*‐stacking interaction occurs with a distance of 4.115 Å so this can be considered weak binding considering typical ranges observed for such interactions.^[^ ^17^ ^]^ The structure of **3** was previously reported^[^ ^18^ ^]^ and, as such, was not explored further in this study.

**Figure 5:**
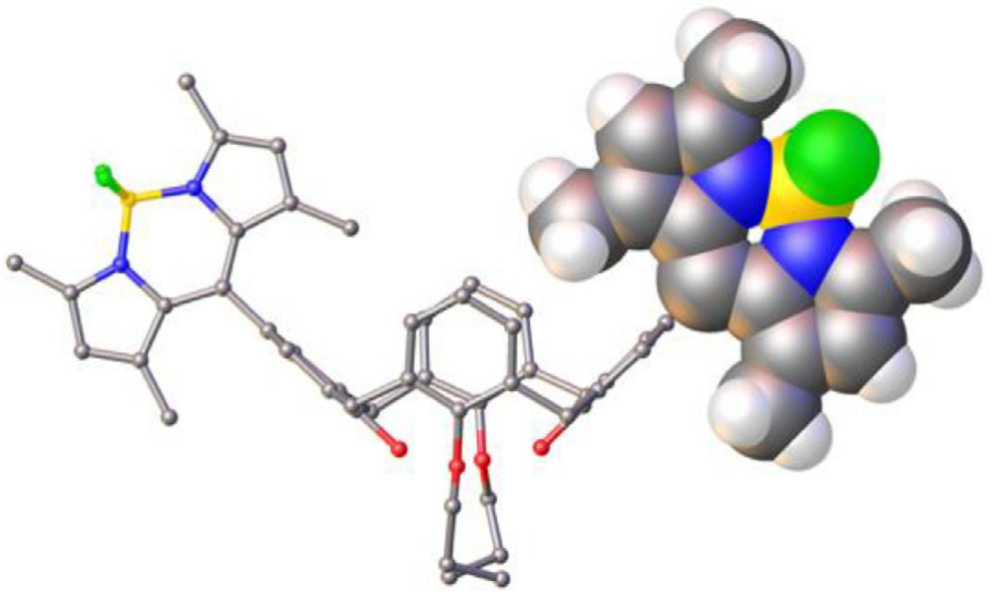
Single crystal X‐ray structure of **4** showing C[4] cavity occupation (in partial space‐filling representation) by the methyl groups that remain locked orthogonally even at high temperature, thus blocking H:G complexation.

Good quality single crystals of **4** were obtained by slow evaporation from DCM, and structural analysis provided a view of the four methyl groups of the BODIPY fragments locked orthogonally in the C[4] cavity (Figure 5), as previously reported in the solution phase.^[^ ^12^, ^13^, ^14^ ^]^ Variable temperature (VT) NMR studies on compound **4** confirmed that the cavity remains blocked even at temperatures reaching 60 °C (Figures S7–S9). Compound **4** therefore offers a relatively useful route for comparison with the behaviors of **1** and **2**, as it would be unable to enhance the effective concentration through H:G complex formation in exactly the same manner. It should, however, be noted that the tetramethyl‐BODIPY fragments themselves present an inherent cavity‐like environment above the blocked C[4] cavity, though this is clearly disparate to the clefts presented by the host framework in both **1** and **2**. As is the case for **3**, the structure of **5** has been previously reported^[^ ^18^ ^]^ and is not of interest, as the molecule does not contain a cavity.

### 2.3 Photophysical Properties

Photophysical experiments were carried out on compounds **1** – **5** to evaluate their suitability for photosensitization of O_2_ (Table 1). All of the compounds exhibited wavelengths of maximum absorption (*λ*_abs,max_) in chloroform in the region of 497 − 503 nm (Figure S10), therefore compounds **1** – **5** require the use of green light (500 − 510 nm) to generate ^1^O_2_. The emission spectra of **1** – **5** were also measured, revealing emission maxima in the range of 514 − 518 nm (Figures S11–S15). Photoluminescence quantum yield (PLQY) measurements were also performed on the BODIPY dyes revealing that the presence of methyl groups influenced their photophysical properties. For compounds **1–3**, the PLQY was measured to be in the range of 0.1130 − 0.1460 which was considerably lower than the values of 0.7009 and 0.6992 obtained for **4** and **5**, respectively. This increase in the PLQY can be attributed to the methyl groups in **4** and **5** suppressing free rotation of the *meso*‐ phenyl group, which is a pathway for nonradiative relaxation of the excited singlet state, as has been reported by others.^[^ ^19^ ^]^ Similar to other BODIPY dyes, **1** – **5** exhibited high molar attenuation coefficients (ε_m_), indicating that they are highly efficient at absorbing incident light.^[^ ^19^ ^]^

**Table 1:** Photophysical properties of photosensitizers **1 – 5**.

Dye	*λ*_abs,max_/nm	*λ*_em,max_/nm	Stokes’ Shift/nm	PLQY	*ε*_m_/10^3^ × M^−1^ cm^−1^
**1**	497	514	17	0.1460	55.5
**2**	498	514	16	0.1073	78.3
**3**	500	518	18	0.1130	60.1
**4**	503	518	15	0.7009	131.7
**5**	503	516	13	0.6992	89.0

### 2.4 Theoretical Studies

Further studies on **1** – **5** were performed using computational methods. Each of the molecules was optimized using CAM‐B3LYP/6–311G(d,p) in a polarizable continuum chloroform model, starting from crystal structure coordinates (further details in Supporting Information).^[^ ^20^ ^]^ Good structural agreement was found between the crystal structures and computation. The singlet‐triplet energy gaps from the lowest triplet state were calculated as being in the range of 127.7 − 149.1 kJ mol^−1^, indicating that **1** – **5** were suitable for sensitizing oxygen, which requires a transition energy of at least 94.5 kJ mol^−1^.^[^ ^7^ ^]^ The nature of bright S_1_ states for **1** – **5** was also determined using TD‐CAM‐B3LYP/6–311G(d,p) (Figure S16). In each case the bright S_1_ state has a very similar absorption and emission profile, and the nature of this state involves only localized ππ* excitation on the BODIPY moieties.

### 2.5 Photocatalytic Studies

**Figure 6:**
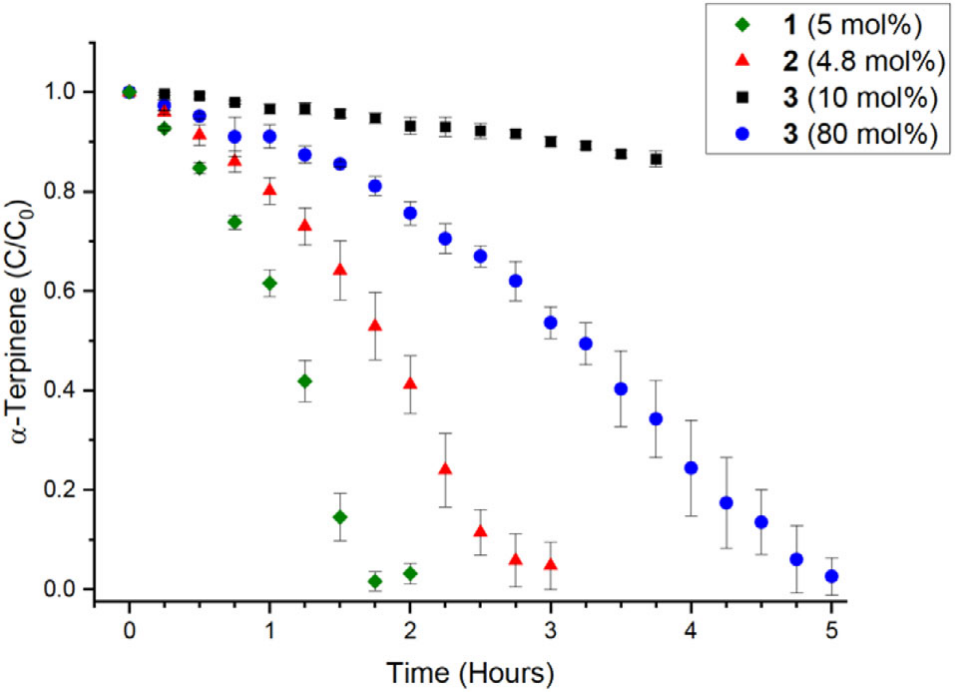
Plot of *α*‐terpinene consumption as a function of time in the presence of photosensitizers **1** – **3** at varying mol% ratios (inset), performed in triplicate.

Compounds **1** – **5** were subsequently employed as photosensitizers in the reaction outlined in Scheme 1, and discussion here focuses on the two sets of photosensitizers separately. Figure 6 shows consumption of *α*‐terpinene over time in the presence of **1** – **3** in varying mol% ratios (inset), with conversion to ascaridole monitored using ^1^H NMR of aliquots taken at regular intervals (e.g., see Figure S17). Inspection of Figure 6 shows complete consumption of *α*‐terpinene in ∼2 hours for **1** at 5 mol%, and ∼3 hours for **2** at 4.8 mol%. In stark contrast, analogous experiments with 3 at 10 mol% show ∼7% consumption after 2 hours and ∼10% after 3 hours. Comparison of these activities shows that the cavities in **1** and **2** promote approximately 28‐ and 10‐fold rate enhancements, respectively. Interestingly, the presence of an additional BODIPY in **2** appears to hinder this process, a fact that may potentially be attributable to restricted substrate access to the cavity through increased steric bulk at the upper‐rim. As compound **3** performed so poorly in a comparable timeframe, it was necessary to increase catalyst loading to near‐stoichiometric amounts (80 mol%) in order to reach full consumption in a timely manner, though this still took ∼5 hours.

**Figure 7:**
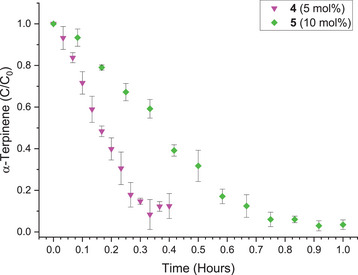
Plot of *α*‐terpinene consumption as a function of time in the presence of photosensitizers **4** and **5** at varying mol% ratios (inset), performed in triplicate.

To further investigate the effect that the presence of the cavity had on the rate of reaction, analogous studies were conducted with **4**, where self‐inclusion of the BODIPY methyl groups renders the cavity inaccessible. To factor out any effect that the introduction of methyl groups might have on the photophysical properties of the photosensitizing group, compound **5** was also synthesized and tested. Examination of Figure 7 shows that, under analogous conditions employed for **1** – **3**, complete consumption of *α*‐terpinene is observed in ∼30 minutes for **4** at 5 mol%, and ∼1 hour for **5** at 10 mol%. The superior performance of both **4** and **5** relative to **1** – **3** can be attributed to the fact that the methyl groups hinder rotation of the phenyl group (see Figures 5 and S7–S9 for **4**), removing a nonradiative relaxation pathway and rendering them more efficient catalysts in general.^[^ ^21^ ^]^ However, when **4** and **5** are compared, it can be seen that only an approximate 2.5‐fold increase in rate is observed. Considering the 10‐fold increase in rate observed when using **2** over **3**, an increase of just 2.5 times suggests that the cavity of **4** is blocked and does not play an influential role in binding to the *α*‐terpinene. As alluded to above, compound **4** presents a *pseudo*‐cavity at the upper‐rim (Figure 5) due to the orientation of the tetramethyl‐BODIPY moieties. This may be a contributing factor which results in the approximate 2.5‐fold rate increase in *α*‐terpinene consumption with **4** relative to **5**. It should also be considered that the overall shape and surface of **4** may also contribute to the observed increase, though this is extremely hard to evaluate; for example, one can envisage other regions of the molecule being capable of forming weak intermolecular interactions with *α*‐terpinene, for example, aromatic rings around the exterior of the calixarene.

In the above experiments we monitored *α*‐terpinene consumption and production of ascaridole in a suitable window of the spectrum using a chloroform suppression sequence; this was undertaken so that integrals could be measured most effectively. We also performed an analogous experiment using CDCl_3_ as a solvent and the readily isolable catalyst **3** to confirm that ascaridole was indeed the major product of the photocatalytic reaction, ruling out significant formation of species such as *p*‐cymene (which is a known product in some similar reactions as previously reported).^[^ ^19^ ^]^

Overnight photobleaching was performed on samples of **1** – **3**, and subsequent photocatalytic runs showed little change in activity (Figures S18–S20). Furthermore, photocatalytic activity of **1** was confirmed to be dependent on the light source, and temperature monitoring of the reaction was also studied over the course of two hours; this showed a relatively small increase of ∼8 °C over 2 hours (Figure S21), which is likely attributable to the nature of the experimental setup; temperature‐controlled housing will be designed and employed in future studies to eliminate this factor.

### 2.6 Competition Experiments

**Figure 8:**
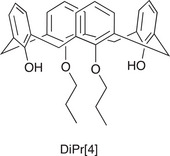
Structure of distal di‐propoxycalix[4]arene, DiPrC[4].

Further experiments were carried out with **1** to consolidate our theory concerning the H:G enhancement over the photocatalytic conversion of *α*‐terpinene to ascaridole. The first experiment in this section involved addition of DiPrC[4] (Figure 8) as a competitive host in the presence of **1**. Consumption of *α*‐terpinene was monitored over time with **1** (5 mol%) and DiPrC[4] present in 1:1 and 1:10 ratios (Figure S22). Though care should be taken in inferring too much in this regard from NMR monitoring (with associated errors), the performance of **1** was not greatly affected by the presence of DiPrC[4]. It is interesting that a lower degree of completion is reached when both calixarenes are present. This experiment was a rough comparison given that the upper‐rim in DiPrC[4] is bare, but also because H:G interactions will be subtly different as a result of alteration to the C[4] framework in general. Despite these differences, this comparison does suggest that the presence of an upper‐rim moiety is important in promoting a level of balanced guest binding (to facilitate turnover) and is something that will be explored in future work with a broader range of upper‐rim photocatalyst moieties present. The reason for consumption reaching only ∼80% with both **1** and DiPrC[4] present remains unclear, but this will also be investigated further as part of future work with this system.

The second experiment involved the addition of *p*‐cymene as a competitive guest for **1** given its structural similarity to *α*‐terpinene. Consumption of 1 equivalent of *α*‐terpinene was monitored over time in the presence of **1** (5 mol%) with either 10 or 25 equivalents of *p*‐cymene added to the reaction mixture (Figure S23). A noticeable reduction in rate was observed in the presence of this competitive guest, with the rate reduction increasing in line with the concentration of *p*‐cymene. It was desirable to further increase the loading of *p*‐cymene to explore if photocatalytic consumption could be inhibited further, but loss in resolution in NMR spectra upon addition of such a large excess of competitive guest rendered this impractical; the challenge associated with this can clearly be seen in Figures S24, S25 when dealing with just 25 equivalents of *p*‐cymene. Although we are unable to monitor this effect in solution at higher concentrations, it is reasonable to assume that further reductions in the rate of consumption of *α*‐terpinene would be observed.

A final experiment was undertaken to demonstrate the importance of covalently tethering the calixarene/BODIPY moieties and hence combining the C[4] cavity directly with the photosensitizer fragment. Consumption of *α*‐terpinene was monitored over time in the presence of **3** (80 mol%), but in this case with DiPrC[4] (40 mol%) added to the reaction mixture (Figure S26). Minimal difference was observed in the rate of *α*‐terpinene consumption relative to the case when **3** was present in an 80 mol% ratio. This clearly indicates that the two moieties must be combined through covalent bond formation at the upper‐rim, again strengthening the argument that the C[4] cavity is key to enhancing the conversion of *α*‐terpinene to ascaridole in this bimolecular reaction.

### 2.7 Binding Studies via Stern‐Volmer Fluorescence Quenching

It should be noted that strong binding in the C[4] cavity was not observed in the solution phase at any point, nor was it expected to be given the design strategy employed. Significant shifts were not observed for guests in NMR spectra, suggesting that binding of *α*‐terpinene in the C[4] cavity is transient and is unlikely to be the rate‐determining step in the overall process. Fluorescence quenching and Stern‐Volmer plots are powerful tools used widely to measure affinity and interactions between molecules. Thus, while this is not the most common practical protocol to obtain a binding constant,^[^ ^22^ ^]^ other examples of the use of the Stern‐Volmer Constant, K_sv_, in parallel with or even in place of binding constant, K_a_, have been reported in recent literature for neutral‐guest sensing with calixarenes as host molecules.^[^ ^23^, ^24^, ^25^, ^26^ ^]^ Thus, to further prove the role of H:G interactions in the enhanced reactivity of the photosensitizers tethered to the C[4] framework, a series of Stern‐Volmer quenching plots were carried out with a fluorimeter for the species of interest, **1**, **2**, **4**, and **5** (as a control), with increasing equivalents of *α*‐terpinene (Figure S27–S30).

The order of magnitude of the K_sv_ (M^−1^) is proportional to the affinity of the molecules measured, and can vary widely from very weak (x10^2^) to strong interactions (x10^10^).^[^ ^23^, ^24^, ^25^, ^26^, ^27^ ^]^ The calculated K_sv_’s reported in summary in Table 2 for **1**, **2**, **4**, or **5**:*α*‐terpinene shows weak to very weak H:G interactions of 6.01 × 10^4^, 5.37 × 10^4^, 1.27 × 10^3^, and 9.72 × 10^2^ M^−1^, respectively. These values may explain the abovementioned lack of observable shift of ^1^H signals in NMR spectra, as they show at best a mild affinity between *α*‐terpinene and the studied species; this may be too low to be detected with other techniques at these concentrations. The obtained K_sv_ values align in the following trend: **1 **> **2 **> **4 **> **5**. However, it is interesting to note that this is not strictly in line with the trend of absolute reactivities obtained in their ^1^O_2_ production. In fact, photosensitizer **5** is a more efficient photosensitizer than **1** or **2** despite the presence of a C[4] cavity, and the best‐performing photosensitizer was found to be **4**. This observation is not unexpected given that **4** and **5** are better photosensitizers due to their inability to undergo nonradiative relaxation of the excited singlet state via free rotation of the *meso*‐phenyl group as outlined above.^[^ ^19^ ^]^

**Table 2:** Summarized results of Stern‐Volmer plots of **1**, **2**, **4**, and **5**.

Photosensitizer	K_sv_ /M^−1^	R^2^	Completion time/ hour
1	60, 090	0.9743	2
2	53, 690	0.9908	3
4	1269.3	0.9830	0.4
5	971.9	0.9686	1 (10 mol%)

The pattern in K_sv_ can instead provide evidence for the H:G interaction, as it aligns with accessibility of the C[4] cavity in **1** and **2**, and not absolute performance. In fact, compound **1** showed the highest K_sv_ value, which can be correlated to its mono‐substitution and hence the least hindered and most accessible cavity of the series. This is followed by **2**, which showed about 10% lower K_sv_ as a consequence of di‐substitution of the C[4]. The K_sv_ of **4** was found to be about 50 times lower than **1** and **2**, which can be explained by its cavity being permanently occupied by methyl groups. Finally, the lowest affinity was found for **5**, which can be correlated to its total lack of a C[4] scaffold/cavity altogether, thus not even presenting a large surface that may be involved in potentially forming weak interactions. This final measurement shows the strongest evidence as a control test, as it is a particularly effective photosensitizer whilst displaying the weakest interaction.

It is therefore clear that the reasoning behind the increment in reactivity is indeed due to H:G interactions, albeit weak, which align with the trend of accessibility of the cavity. Oxidation of the product and egress from the cavity frees up space for occupation by another reactant molecule, a process that is likely driven by a marked change in shape upon moving from *α*‐terpinene to ascaridole.

## 3 Conclusion

To conclude, we have shown that BODIPY photosensitizer moieties tethered to the upper‐rim of calix[4]arene scaffolds enhance the photocatalytic conversion of *α*‐terpinene to ascaridole through the formation of weak H:G interactions. Bis‐functionalization at the upper‐rim hinders catalysis relative to the mono‐functionalized analogue, possibly due to hindering guest access to the C[4] cavity. Superior performance relative to a blank photosensitizer is attributed to enhanced concentration between the substrate and locally generated ^1^O_2_, all of which is reliant upon the presence of a C[4] cavity. Tetramethyl‐BODIPY analogues are efficient photosensitizers due to their inability to rotate and undergo nonradiative relaxation of the excited singlet state. Although this is the case, introducing these moieties directly to the upper‐rim of a C[4] blocks the cavity and precludes guest occupation and only a 2.5‐fold increase is observed compared to the relative blank photosensitizer.

This work opens up several avenues for future investigation, examples of which include the incorporation of these photosensitizing host molecules into polymers/onto solid supports through lower‐rim functionalization, exploration of selectivity in terpenes of different shapes and sizes (as well as C[*n*]s of varied ring sizes). Further alteration to the C[*n*] upper‐rim will also be undertaken with a view to tuning cavity shape, size, and functionality. An example here would be the introduction of extended tetramethyl‐BODIPY moieties to exploit the C[4] cavity whilst simultaneously benefiting from the increased efficiency of the catalytic fragment of the host. The ultimate goal of this work will be to demonstrate selectivity in tandem with enhanced effective concentration, finding the perfect balance between all of the factors in play to afford the optimum host for a particular guest. Results from these new areas of investigation will be reported in due course.

## Conflict of Interest

The authors declare no conflicts of interest.

## Supporting information



Supporting Information

Supporting Information

## Data Availability

The data that support the findings of this study are available in the supplementary material of this article.
