# Supplementary material for: Host Cavities Enhance the Photocatalytic Conversion of α‐Terpinene to Ascaridole Under Visible‐Light Irradiation
Source: Chemistry. 2025 Sep 2;31(55):e02113. doi: 10.1002/chem.202502113 (PMC12498068; doi:10.1002/chem.202502113)
Supplement: Supplementary file 1 — Supporting Information [file CHEM-31-e02113-s001.docx]

**Host cavities enhance the photocatalytic conversion of *α*-terpinene to ascaridole under visible-light irradiation**

**Electronic Supplementary Information**

**Table of Contents**

1. **Synthetic Details**
   1. Synthesis of Key Starting Materials …………………………………………………….….………………………..…2
   2. Synthesis of  **1 – 5** ..…………….………………………………………………………………..………………………………3
2. **Single Crystal X-Ray Structures** ………………………………..……..……………………………..……..………………………..9
3. **Variable Temperature NMR of 4** ………………………………..……..……………………………..……..……………………13
4. **Photophysical Properties of 1 – 5** ………………………………………………………………………………………………….15
   1. UV-Vis Absorption Spectra …………………………………………………………………….…………….……..…….15
   2. Molar Attenuation Coefficients ………….……………………………………..………………………………..…….15
   3. Emission Spectra and PLQY ………………………………………………..……………………………..………………16
5. **Computational Studies** …..……………………….……..……………………………..……..…………………………..……...…20
6. **Photosensitisation ^1^O_2_ Generation and Reaction with 𝛂-Terpinene** ..………………………………….….……22
   1. Procedures .……………………………………………………………………………….…………………….……………….22
   2. Overnight Bleaching Tests ….…………………….…………………….…………………….………………………….23
   3. Control Experiments ..……………………………………………………….……………….……………………………..25
7. **Binding studies via Stern-Volmer Fluorescence Quenching**…...………..……..……….…..……………..……….31
8. **Spectroscopic Characterisation of Synthesised Products** …...………..……..……………..………….…..……….36
9. **References** ….……………………………………………………….………………………….……………………………..………….…40

**1. Synthetic Details**

As outlined in the manuscript, some compounds presented in the paper (including their intermediates) have been reported previously in the literature. Where this is the case below, we have included citations but also comments in relation to reported literature yield and any differences in synthetic approach employed.

Compounds **1** and **2** were synthesised using mono- or di-formyl calix[4]arenes as shown in Scheme S1.^[S1-S4]^

**Scheme S1.** Synthesis of compounds **1** and **2** starting from calix[4]arene and proceeding *via* lower-rim distal di-alkylation with iodopropane in the presence of base,^[S5]^ mono- or di-formylation at the upper-rim using dichloromethyl methyl ether and titanium tetrachloride,^[S6]^ and subsequent reaction with pyrrole in the presence of trifluoroacetic acid, 2,3-dichloro-5,6-dicyano-1,4-benzoquinone (DDQ), triethylamine and then boron trifluoride diethyl etherate.

**1.1 Synthesis of Key Starting Materials**

**25,27-Dipropoxycalix[4]arene ^[S5]^**

Calix[4]arene (10.003 g, 23.565 mmol) was suspended in acetonitrile (500 mL). 1-iodopropane (15.69 g, 92.28 mmol) and potassium carbonate (13.026 g, 94.24 mmol) were added in rapid succession. The resultant solution was heated at reflux for 24 h. The solution was cooled to room temperature, dried over MgSO_4_ and rotary evaporated to dryness. This produced a white solid to which 10 v/v % HCl (250 mL) and dichloromethane (250 mL) were added. The organic product was separated and washed with water (2 x 250mL) before being rotary evaporated to dryness. White crystals of product were crystallised from a 2 layer system of dichloromethane : methanol (7.313  g, 61 %). ^1^H NMR (300 MHz, CDCl_3_) δ_H_ 8.31 (s, 2H), 7.07 (d, J = 7.47 Hz, 4H), 6.93 (d, J = 7.53 Hz, 4H), 6.75 (dd, J = 7.96, 7.10 Hz, 2H), 6.65 (m, 2H), 4.34 (d, J = 12.93 Hz, 4H), 3.99 (t, J = 6.30 Hz, 4H), 3.39 (d, J = 12.93 Hz, 4H), 2.08 (m, 4H), 1.33 (t, J = 7.42 Hz, 6H).

Synthetic procedure followed was from reference [S5]. The yield obtained was similar to literature values.

**5-Formyl-25,27-Dipropoxycalix[4]arene ^[S2-S3,S6]^**

25-27-di-Propoxycalix[4]arene (1.000 g, 1.97 mmol) was dissolved in dry dichloromethane (45 mL) under a N_2_ atmosphere. Dichloromethyl methyl ether (0.407 g, 3.59 mmol) was added and the resultant mixture stirred for 15 minutes at room temperature. After this time, TiCl_4_ (1.47 g, 7.75 mmol) was added and the stirring continued for another 45 minutes before dichloromethane (5 mL) and water (30 mL) were added sequentially. The organic phase was collected and washed with sat. NaHCO_3_ (2 x 30 mL) causing decolourisation of the organic layer. The resultant organic phase was dried over Na_2_SO_4_ and rotary evaporated to dryness producing a yellow solid. The product was then purified by flash column chromatography (acetone : dichloromethane (2.5 : 97.5)) yielding an off-white powder (0.474 g, 44.8 %). ^1^H NMR (300 MHz, CDCl_3_) δ_H_ 9.79 (s, 1H), 9.31 (s, 1H), 8.26 (s, 1H), 7.63 (s, 2H), 7.06 (d, J = 7.47 Hz, 2H), 6.95 (m, 4H), 6.77 (t, J = 7.55 Hz, 2H), 6.65 (t, J = 7.47 Hz, 1H), 4.31 (dd, J = 13.03, 2.79 Hz, 4H), 4.00 (m, 4H), 3.44 (dd, J = 27.39, 13.07 Hz, 4H), 2.08 (m, J = 6.95 Hz, 4H), 1.33 (t, J = 7.38 Hz, 6H).

This procedure was adapted from one for analogous compounds in references S2, S3 and S6. Yields are not directly comparable due to dissimilarity in structure between reference compounds and the product. The primary difference between the literature procedure and that used herein is the use of TiCl_4_ instead of SnCl_4_. This was due to overall yield improvement.

**5,17-Di-formyl-25,27-Dipropoxycalix[4]arene ^[S3,S6]^**

25-27-di-Propoxycalix[4]arene (4.315 g, 8.483 mmol) was dissolved in dry dichloromethane (250 mL) under a N_2_ atmosphere. Dichloromethyl methyl ether (3.57 g, 31.03 mmol) was added and the resultant mixture stirred for 15 minutes at room temperature. After this time, TiCl_4_ (12.98 g,  68.40 mmol) was added and the stirring continued for another 45 minutes before dichloromethane (40 mL) and water (110 mL) were added sequentially. The organic phase was collected and washed with sat. NaHCO_3_ (3 x 50 mL). The resultant organic phase was dried over Na_2_SO_4_ and rotary evaporated to dryness producing the desired product as an off-white solid (4.79 g, 91% yield). ^1^H NMR (300 MHz, CDCl_3_) δ_H_ 9.72 (s, 2H), 9.18 (s, 2H), 7.56 (s, 4H), 6.91 (d, J = 7.50 Hz, 4H), 6.73 (m, 2H), 4.24 (d, J = 13.12 Hz, 4H), 3.95 (t, J = 6.20 Hz, 4H), 3.44 (s, J = 13.21 Hz, 4H), 2.02 (sex, J = 6.95 Hz, 4H), 1.26 (t, J = 7.43 Hz, 6H).

This procedure was adapted from one for analogous compounds in references S3 and S6. Yields are not directly comparable due to dissimilarity in structure between reference compounds and the product. The primary difference between the literature procedure and that used herein is the use of TiCl_4_ instead of SnCl_4_. This was due to overall yield improvement.

**1.2 Synthesis of Photosensitisers**

**5-Dipyrromethyl-25,27-*di*-propoxycalix[4]arene ^[S2]^**

5-Formyl-25-27-*di*-propoxycalix[4]arene (2.210 g, 4.12 mmol) added to an excess of pyrrole (2.76 g, 41.1 mmol) under an N_2_ atmosphere. A catalytic amount of trifluoroacetic acid (3 drops) was added and the resultant mixture stirred for 25 minutes. After this time, sodium hydroxide 1M (10 mL) was added to the solution before the mixture was diluted with water (100 mL) and dichloromethane (100 mL). The organic layer was collected before washing with water (2 x 100 mL). The organic layer was then placed under high vacuum to help remove any remaining pyrrole, producing a tacky black solid. This was purified using column chromatography (ethyl acetate : dichloromethane : pet. Ether: triethyl amine (2 : 1 : 7 : trace)), followed by a second column in acetone : pet. ether : ethyl acetate (1 : 3 : 1) producing a dark orange semi-crystalline product (0.7468 g, 28%). ^1^H NMR (300 MHz, CDCl_3_) δ_H_ 8.41 (s, 1H), 8.38 (s, 1H), 7.76 (bs, 2H), 6.99 (d, J = 7.50 Hz, 2H), 6.90 (d, J = 1.68 Hz, 1H), 6.88 (d, J = 1.74 Hz, 1H), 6.80 (m, 2H), 6.77 (d, J = 1.71 Hz, 1H), 6.70 (t, J = 7.48 Hz, 2H), 6.59 (m, 3H), 6.09 (q, J = 2.87 Hz, 2H), 5.88 (m, 2H), 5.28 (bs, 1H), 4.24 (dd, J = 12.79, 10.87 Hz, 4H), 3.90 (t, J = 6.27 Hz, 4H), 3.28 (dd, J = 25.68, 12.84 Hz, 4H), 2.00 (m, 4H), 1.25 (t, J = 7.41 Hz, 6H).

This procedure was adapted from one for analogous compounds in reference S2. Yields are not directly comparable due to dissimilarity in structure between compounds in the reference and the product in this case.

**5,17-*di*-Dipyrromethyl-25,27-*di*-propoxycalix[4]arene ^[S1]^**

5,17-*di*-Formyl-25,27-*di*-propoxycalix[4]arene (0.998 g, 1.77 mmol) was suspended in an excess of pyrrole (11.60 g, 173 mmol) before 5 drops of trifluoroacetic acid were added. The resultant reaction mixture was stirred for 35 minutes at room temperature. After this time, 0.1M NaOH (30 mL) was added. The mixture was washed with ethyl acetate (1x60 mL) and the organic product was collected. The aqueous phase was extracted with ethyl acetate (2x30 mL) and the organic products were combined before being dried over Na_2_SO_4_. The product was rotary evaporated to produce a red oil which was purified using column chromatography (ethyl acetate : dichloromethane (2 : 1)) followed by a second column (ethyl acetate : dichloromethane : pet. Ether (6 : 2 : 2)) to give an brown-yellow semi-crystalline product (0.8283 g, 59 %). ^1^H NMR (300 MHz, CDCl_3_) δ_H_ 8.24 (bs, 2H), 7.90 (m, 4H), 6.64 (m, 8H), 6.51 (dd, J = 8.07, 6.97 Hz, 2H), 6.38 (m, 4H), 5.91 (q, J = 2.81 Hz, 4H), 5.68 (m, 4H), 5.07 (s, 2H), 4.07 (d, J = 12.75 Hz, 4H), 3.73 (t, J = 6.14 Hz, 4H), 3.08 (d, J = 12.93 Hz, 4H), 1.85 (m, 4H), 1.11 (t, J = 7.38 Hz, 6H).

Literature procedure followed from reference S1. Reported yield of 59% was lower with respect to the literature value of 85%.

**5-BODIPY-25,27-*di*-Propoxycalix[4]arene, 1 ^[S2]^**

5-Dipyrromethyl-25,27-Dipropoxycalix[4]arene (746.8 mg, 1.144 mmol) was dissolved in dry toluene (5 mL). 2,3-Dichloro-5,6-dicyano-1,4-benzoquinone (259.2 mg, 1.142 mmol) was added and the solution stirred for 15 minutes. An excess of triethylamine (3.63 g, 35.9 mmol) and boron trifluoride diethyl etherate (4.29 g, 30.2  mmol) were added and stirred for a further 21 hours. The resultant solution was diluted with dichloromethane (200 mL) and washed with once with H_2_O (100 mL). The aqueous layer was then extracted with dichloromethane (100 mL). This was combined with the previous organic phase, and the resultant solution was washed with H2O (2 x 200 mL). The organic phase was dried with Na_2_CO_3_ and solvent was removed by rotary evaporation to give an orange oil, which was purified using column chromatography (Sole eluent dichloromethane) and recrystallized from dichloromethane  : pet ether 40-60 (Vapour diffusion) to give an orange crystalline product (398 mg, 50 %). ^1^H NMR (300 MHz, CDCl_3_) δ_H_ 9.18 (s, 1H), 8.47 (s, 1H), 7.90 (bs, 2H), 7.35 (s, 2H), 7.08 (d, J = 7.50 Hz, 2H), 6.98 (m, 6H), 6.84 (t, J = 7.53 Hz, 2H), 6.66 (t, J = 7.47 Hz, 1H), 6.54 (dd, J = 4.16, 1.82 Hz, 2H), 4.37 (dd, J = 15.34, 12.97 Hz, 4H), 4.02 (m, 4H), 3.45 (t, J = 12.94 Hz, 4H), 2.11 (m, 4H), 1.36 (t, J = 7.40 Hz, 6H), ^13^C NMR (75.5 MHz, CDCl_3_) δ_C_ 156.79 (C_q_), 153.45 (C_q_), 151.92 (C_q_), 148.22 (C_q_), 142.75 (CH), 134.84 (C_q_), 133.77 (C_q_), 132.80 (C_q_) 131.62 (CH), 131.42 (CH), 129.49 (CH), 128.77 (CH), 128.54 (CH), 127.89 (C_q_), 125.61 (CH), 119.11 (CH), 117.94 (CH), 78.58 (CH_2_), 31.53 (CH_2_), 31.45 (CH_2_), 23.52 (CH_2_), 10.96 (CH_3_),  ^11^B (96.3 MHz, CDCl_3_, δ): 0.32 (t, J = 28.97 Hz), ^19^F (282.4 MHz, CDCl_3_, δ): ‑145.19 (1:1:1:1 q, J = 29.06 Hz); IR ν (cm^-1^) 3331 (w, OH), 2928 (s, C-H) MS calculated 698.31, found 721.30 [M+1Na].

This procedure was adapted from that for analogous compounds in reference S2. Yields are not directly comparable due to dissimilarity in structure between reference and presented product.

**5,17-*di*-BODIPY-25,27-*di*-Propoxycalix[4]arene, 2^[S1]^**

5,17-*di*-Dipyrromethyl-25,27-*di*-propoxycalix[4]arene (781.3 mg, 0.980 mmol) was dissolved in dry toluene (40 mL). To this, 2,3-dichloro-5,6-dicyano-1,4-benzoquinone (447.6 mg, 1.97 mmol) was added and the resulting solution stirred at room temperature for 15 minutes. Excess triethylamine (3.63 g, 35.9 mmol) and boron trifluoride diethyl etherate (5.75 g, 40.5 mmol) were then added dropwise and stirring continued at room temperature for a further 4 hours. Dichloromethane (200 mL) and water (100 mL) were then added to the reaction mixture. Stirring was continued for 5 minutes before the organic layer was separated. This was washed with a brine solution (2 x 200 mL). The organic product was dried over Na_2_CO_3_ before being rotary evaporated to dryness and purified by column chromatography (dichloromethane (100%)) to produce orange crystalline product (176 mg, 20 %). ^1^H NMR (300 MHz, CDCl_3_) δ_H_ 9.21 (s, 2H), 7.91 (bs, 4H), 7.40 (s, 4H), 7.02 (m, 8H), 6.92 (dd, J = 8.39, 6.56 Hz, 2H), 6.56 (dd, J = 4.17, 1.98 Hz, 4H), 4.41 (d, J = 13.03 Hz, 4H), 4.06 (t, J = 6.15 Hz, 4H), 3.52 (d, J = 13.30 Hz, 4H), 2.14 (m, 4H), 1.39 (t, J = 7.40 Hz, 6H), ^13^C NMR (75.5 MHz, CDCl_3_) δ_C_ 156.00 (C_q_), 150.92 (C_q_), 146.98 (C_q_), 141.82 (CH), 133.71 (C_q_), 131.84 (C_q_), 130.86 (CH), 130.32 (CH), 127.27 (C_q_), 124.71 (CH), 123.98 (C_q_), 117.01 (CH), 77.73 (CH_2_), 76.20 (CH), 30.55 (CH_2_), 22.53 (CH_2_), 10.00 (CH_3_), ^11^B (96.3 MHz, CDCl_3_, δ): 0.32 (t, J = 28.87), ^19^F (282.4 MHz, CDCl_3_, δ­): -145.18 (1:1:1:1 q, J = 28.90 Hz ); IR ν (cm^-1^) 3199 (w, OH), MS calculated 888.36, found 911.35 m/z [M+Na].

Literature procedure followed from reference S1. Reported yield of 20% was lower with respect to literature value of 52%.

Compound **3** was synthesised according to the procedure reported by Baruah *et al.*^[S7]^ and as shown in Scheme S2.

^^

**Scheme S2.** Synthesis of compound **3** *via* reaction of 4-hydroxybenzaldehyde with pyrrole, 2,3-dichloro-5,6-dicyano-1,4-benzoquinone, triethylamine and then boron trifluoride diethyl etherate.^[S7]^

**5-(4-Hydroxyphenyl)dipyrromethane, 3 ^[S7]^**

4-Hydroxybenzaldehyde (1.552 g, 12.71 mmol) was dissolved in an excess of neat pyrrole (6.769 g, 100.9 mmol). N_2_ was bubbled through the solution for 10 minutes before a catalytic amount of trifluoroacetic acid was added. The resultant solution was then stirred under N_2_ for 25 minutes. 0.1M NaOH (10 mL) was added. The resultant mixture was diluted in ethyl acetate (30 mL) and stirred for 1 hour. The organic layer was separated and the aqueous layer extracted with ethyl acetate (3 x 30 mL). Organic material was combined and dried over MgSO_4_ before the solvent was removed by rotary evaporation to produce a viscous black oil. The organic product was then dried under high vacuum for 8 hours before being heated at 300 ^o^C to produce a black resin. This product was purified by column chromatography (ethyl acetate : dichloromethane : pet. Ether (5 : 1 : 4)). Product collected as an off white powder (1.031 g, 34 %). ^1^H NMR (300 MHz, CDCl_3_) δ_H_ 10.46 (s, 2H), 9.17 (s, 1H), 6.96 (m, 2H), 6.66 (m, 2H), 6.58 (m, 2H), 5.88 (m, 2H), 5.63 (m, 2H), 5.23 (bs, 1H), ^13^C NMR (75.5 MHz, CDCl_3_) δ_C_ 129.52 (CH), 117.20 (CH), 115.39 (CH), 108.25 (CH), 107.01 (CH), 43.06 (CH), 21.05 (C_q_), 14.12 (C_q_), 1.02 (C_q_).

Literature procedure followed from reference S7. Reported yield was not comparable to literature values as this compound was not detailed in the original study.

**4-BODIPY-Phenol, 3 ^[S7]^**

4-Hydroxy-Dipyrromethyl benzene (1.982 g, 8.091 mmol) was dissolved in dry dichloromethane (50 mL) and 2,3-dichloro-5,6-dicyano-1,4-benzoquinone (1.8644 g, 8.213 mmol) was added and the resultant reaction mixture stirred for 15 minutes. After this time, excess triethylamine (7.26 g, 71.7 mmol) and excess boron trifluoride diethyl etherate (11.5 g , 81.0 mmol) were added dropwise before stirring continued for a further 3 hours. The reaction was quenched by the addition of water (200 mL) and diluted with dichloromethane (1 L). Organic product separated and washed with water (2 x 200 mL) before being collected and dried with Na_2_CO_3_ and rotary evaporated. The product was purified through dry loaded gradient column chromatography (pet. ether : acetone (3 : 2) to (1 : 1)) to produce orange crystalline needles (152 mg, 7 %). ^1^H NMR (300 MHz, CDCl_3_) δ_H_ 7.92 (bs, 2H), 7.49 (m, 2H), 6.97 (m, 4H), 6.55 (dd, J = 4.16, 1.55 Hz, 2H), 5.42 (bs, 1H), ^13^C NMR (75.5 MHz, CDCl_3_) δ_C_ 158.35 (C_q_), 147.34 (C_q_), 143.53 (CH), 134.82 (C_q_), 132.61 (CH), 131.41 (CH), 126.45 (C_q_), 118.38 (CH), 115.60 (CH), ^11^B NMR (96.3 MHz, CDCl_3_): δ_B_ 0.31 (t, J = 28.93 Hz), ^19^F NMR (282.4 MHz, CDCl_3_,­) δ_F_ - 144.78 (1:1:1:1 q, J = 28.99 Hz); IR ν (cm^-1^) 3467 (s, OH).

Literature procedure followed from reference S1. Reported yield of 7% was lower with respect to literature value of 64%.

Compound **4** was synthesised according to the procedure reported by Kim *et al.*^[S4]^ and as shown in Scheme S3.

**Scheme S3.** Synthesis of compound **4** *via* reaction of 5,17-diformyl-25,27-dihydroxy-26,28-dipropyloxycalix[4]arene with 2,4-dimethylpyrrole in the presence of trifluoroacetic acid, 2,3-dichloro-5,6-dicyano-1,4-benzoquinone, triethylamine and then boron trifluoride diethyl etherate.^[S4]^

**5,17-Di(Me_4_BODIPY)-25,27-dipropyloxycalix[4]arene, 4 ^[S2,S4]^**

A round bottom flask was charged with 5,17-diformyl-25,27-dihydroxy-26,28-dipropyloxycalix[4]arene (2.541 g, 4.5 mmol) and DCM (100 mL). 2,4-Dimethylpyrrole (1.83 mL, 18.0 mmol) was added along with three drops of trifluoroacetic acid. The mixture was then stirred overnight under nitrogen. Following this a solution of 2,3-dichloro-5,6-dicyano-1,4-benzoquinone (2.043 g, 9.0 mmol) was dissolved in DCM (200 mL) with cooling from an ice bath. This solution was then slowly added to the reaction mixture with cooling and then the mixture allowed to stir for 30 minutes. Following this, triethylamine (10 mL) and boron trifluoride diethyl etherate (10 mL) were added to the reaction and then the mixture left to stir under nitrogen overnight. The solution was then diluted with DCM and washed with brine (6 x 100 mL). The organic phase was then dried over Na­_2_SO_4_ and then the crude black product passed through a silica gel plug using DCM to remove baseline impurities. The crude product was then purified by column chromatography using DCM:Pet. Ether 40-60 ^o^C 2.5:1 v/v as the eluent followed by recrystallisation using DCM/MeOH. This yielded an orange crystalline solid with a green lustre (23 mg, 0.5%). ^1^H NMR (300 MHz, CDCl_3_) δ_H_ 8.46 (s, 2*H*), 6.99 (s, 4*H*), 6.85 (d, 4*H*_,_ ^3^J_HH_ = 7.6 Hz), 6.67 (t, 2*H,* ^3^J_HH_ = 7.6 Hz), 6.00 (s, 2*H*), 5.90 (s, 2*H*), 4.38 (d, 4*H*, ^2^J_HH_ = 13.0 Hz), 4.02 (t, 4*H*, ^3^J_HH_ = 6.2 Hz), 3.40 (d, 4*H,* ^2^J_HH_ = 13.0 Hz), 2.57 (s, 6*H*), 2.55 (s, 6*H*), 2.12 (m, 4*H*), 1.59 (s, 6*H*), 1.37 (s, 6*H*, ^3^J_HH_ = 7.4 Hz), 0.75 (s, 6*H*). ^13^C NMR (75.5 MHz, CDCl_3_) δ_C_ 155.3 (C), 158.8 (C), 153.6 (C), 151.6 (C), 143.4 (C), 143.2 (C), 142.4 (C), 133.4 (C), 132.1 (C), 131.8 (C), 129.6 (C), 128.7 (CH), 127.7 (CH), 125.5 (C), 124.8 (CH), 120.9 (CH), 120.9 (CH), 78.6 (CH_2_), 31.0 (CH_2_), 23.5 (CH_2_), 14.9 (CH_3_), 14.6 (CH_3_), 14.5 (CH_3_), 13.2 (CH_3_). ^11^B NMR (96.3 MHz, CDCl_3_) δ_B_ 0.84 (t, 2*B*, ^1^J_BF_ = 31.6 Hz). ^19^F NMR (282.4 MHz, CDCl_3_) δ_F_ -146.30 (q, 4*F*, ^1^J_BF_ = 31.8 Hz). UV-Vis (nm) λ_max_ (CHCl_3_) 503 nm. IR v (cm^-1^) 3341 (w, OH), 1713 (w, C=N), 1540 (m, B-F). HRMS (ESI) Calcd. for C_60_H_62_B_2_F_4_N_4_O_4_ [M+H^+^] 1000.4972. Found 1001.4966.

This procedure was adapted from one for analogous compounds in references S2 and S4. Reported yield of below 1% was lower than the literature yield of 14%.

**4,4-Difluoro-8-(4-hydroxyphenyl)-1,3,5,7-tetramethyl-4-bora-3a,4adiaza-s-indacene, 5 ^[S1]^**

A round bottom flask was charged with p-hydroxybenzaldehyde (733 mg, 6.0 mmol) and DCM (200 mL). 2,4-Dimethylpyrrole (1.412 g, 1.528 mL, 15.0 mmol) was added along with three drops of trifluoroacetic acid. The mixture was then stirred overnight under nitrogen. Following this a solution of 2,3-dichloro-5,6-dicyano-1,4-benzoquinone (2.724 g, 12.0 mmol) was dissolved in DCM (200 mL) with cooling from an ice bath. This solution was then slowly added to the reaction mixture with cooling and then the mixture allowed to stir for 30 minutes. Following this, triethylamine (10 mL) and boron trifluoride diethyl etherate (10 mL) were added to the reaction and then the mixture left to stir under nitrogen overnight. The solution was then diluted with DCM and washed with brine (6 x 100 mL). The organic phase was then dried over Na­_2_SO_4_ and then the crude black product passed through a silica gel plug using DCM to remove baseline impurities. The crude product was then purified by column chromatography using DCM as the eluent. Final product was obtained as red crystals with a green luster (260 mg, 13%). ^1^H NMR (300 MHz, CDCl_3_, 25.0 ^o^C) δ_H_ 7.14 (m, 2 *H*), 6.95 (m, 2 *H*), 6.00 (s, 2 *H*), 4.90 (s, 1 *H*), 2.55 (s, 6 *H*), 1.45 (s, 6 *H*). ^13^C NMR (75.5 MHz, CDCl_3_, 25.0 ^o^C) δ_C_ ­156.2 (C), 155.4 (C), 143.1 (C), 141.7 (C), 131.9 (C), 129.5 (CH), 127.4 (C), 121.2 (CH), 116.1 (CH), 14.6 (CH_3_), 14.6 (CH_3_). ^11^B NMR (96.3 MHz, CDCl_3_ 25.0 ^o^C) δ_B_ 0.80 (t, ^1^J_BF_ = 32.9 Hz). ^19^F NMR (75.5 MHz, CDCl_3_, 25.0 ^o^C) δ_F_ -146.2 (q, ^1^J_BF_ = 32.9 Hz). IR (cm^-1^) 3398 (m, O-H str.). UV-Vis (CHCl_3_) λ_max_ (nm) 503. HRMS (ESI) Calcd. for C_19_H_19_BF_2_N_2_O [M+H^+^] 341.1637. Found 314.1628.

Literature procedure adapted from analogous moiety in reference [S1].

**2. Single Crystal X-ray Structures**

As outlined in the manuscript, crystallisation of **1** and **2** was attempted by vapor diffusion from a range of solvents with varied size and character (toluene, DCM, chloroform or mesitylene), in all cases using petroleum ether as a counter solvent. Unfortunately both **1** and **2** were insoluble in *α*-terpinene so precluded any direct observation of cavity occupation, and any attempts to include this in a crystallization mixture were unsuccessful. Single crystals suitable for diffraction studies were obtained for **1** from toluene or mesitylene, and for **2** from DCM and toluene (two morphologies). Compounds **3** and **4** were only crystallized from DCM. In the case of compound **3** this crystallized as a hydrate upon slow evaporation, matching entry ESOPOT in the Cambridge Structural Database.^[S8]^ Data for **1⋅Tol**, **1⋅Mes** and **2⋅Tol (side-on)**were collected on a Bruker Apex II diffractometer operating with synchrotron radiation (Advanced Light Source, station 12.2.1) and a PHOTON II detector. Data for **2⋅DCM** were collected on an in-house Bruker Apex II CCD diffractometer. Data for **2⋅Tol (vertical)** and **4⋅DCM**were collected on an in-house Bruker D8 Venture diffractometer operating with a PHOTON III detector.

**Structure of 1⋅Tol**

**Crystal Data for 1⋅Tol (CCDC 1962277, *M*=1489.30 g/mol):** monoclinic, space group *P*2_1_/*n* (no. 14), *a* = 23.0697(8) Å, *b* = 13.5992(5) Å, *c* = 25.6235(8) Å, *β* = 106.0740(10)°, *V*= 7724.6(5) Å^3^, *Z* = 4, *T* = 100(2) K, μ(Synchrotron), *Dcalc* = 1.281 g/cm^3^, 141126 reflections measured (2.792° ≤ 2Θ ≤ 62.162°), 24723 unique (*R*_int_ = 0.0626, R_sigma_ = 0.0475) which were used in all calculations. The final *R*_1_ was 0.0452 (I > 2σ(I)) and *wR*_2_ was 0.1190 (all data).

**
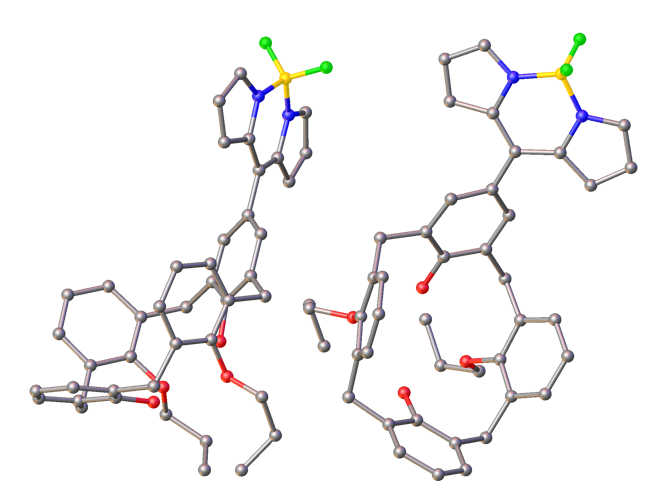
**

**Figure S1.** Single crystal X-ray structure of **1⋅Tol** with H atoms omitted for clarity.

**Structure of** **1⋅Mes**

**Crystal Data for 1⋅Mes (CCDC 1962278, *M*=1517.36 g/mol):** monoclinic, space group *P*2_1_/*n* (no. 14), *a* = 23.2081(10) Å, *b* = 13.6694(6) Å, *c* = 25.4832(12) Å, *β* = 105.854(2)°, *V*= 7776.8(6) Å^3^, *Z* = 4, *T* = 100(2) K, μ(Synchrotron), *Dcalc* = 1.296 g/cm^3^, 131113 reflections measured (3.408° ≤ 2Θ ≤ 62.774°), 23705 unique (*R*_int_ = 0.0586, R_sigma_ = 0.0458) which were used in all calculations. The final *R*_1_ was 0.0582 (I > 2σ(I)) and *wR*_2_ was 0.1758 (all data).

**
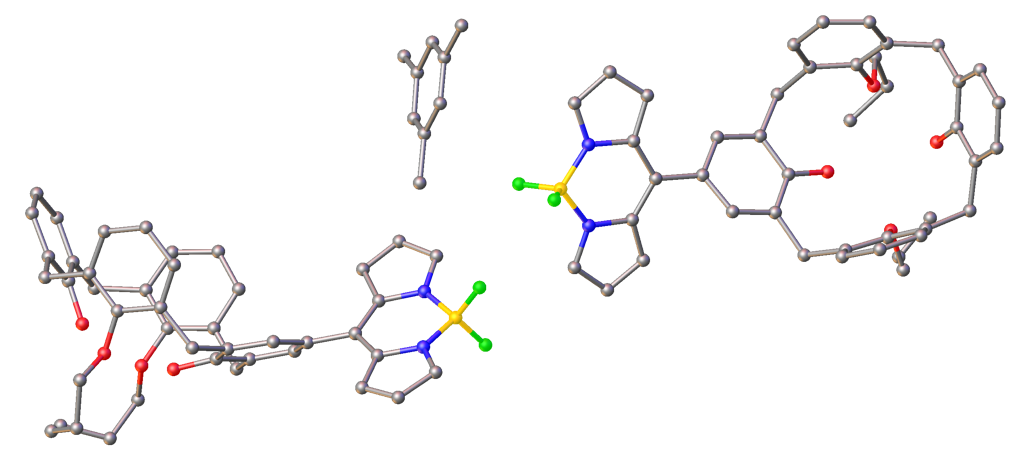
**

**Figure S2.** Single crystal X-ray structure of **1⋅Mes** with H atoms omitted for clarity.

**Structure of 2⋅DCM**

**Crystal Data for 2⋅DCM (CCDC 1962279, *M*=1058.40 g/mol):** monoclinic, space group *P*2_1_/*n* (no. 14), *a* = 14.8995(7) Å, *b* = 15.9788(7) Å, *c* = 21.3035(9) Å, *β* = 101.995(2)°, *V*= 4961.1(4) Å^3^, *Z* = 4, *T* = 100(2) K, μ(MoKα) = 0.306 mm^-1^, *Dcalc* = 1.417 g/cm^3^, 84274 reflections measured (3.06° ≤ 2Θ ≤ 54.208°), 10940 unique (*R*_int_ = 0.0392, R_sigma_ = 0.0273) which were used in all calculations. The final *R*_1_ was 0.0761 (I > 2σ(I)) and *wR*_2_ was 0.2428 (all data).

**
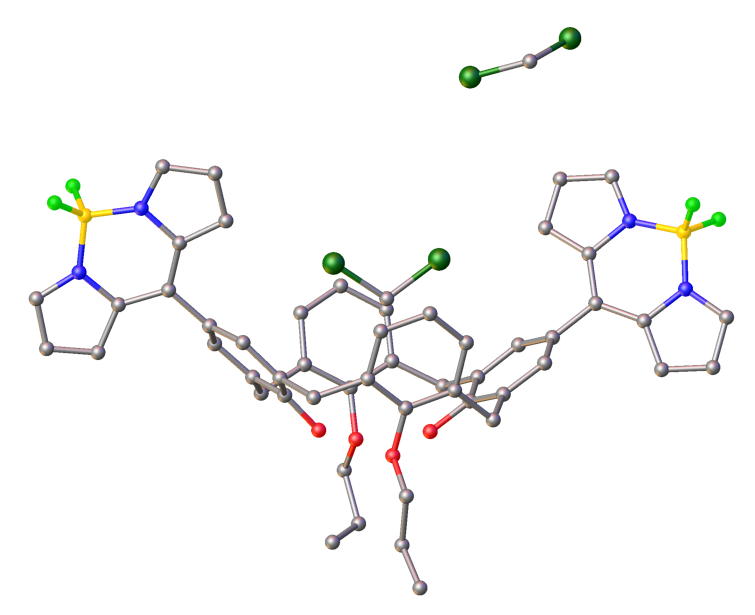
**

**Figure S3.** Single crystal X-ray structure of **2⋅DCM** with H atoms omitted for clarity.

**Structure of 2⋅Tol (vertical)**

**Crystal Data for 2⋅Tol (vertical) (CCDC 1962280, *M*=980.68 g/mol):** orthorhombic, space group *Pbca* (no. 61), *a* = 19.8667(3) Å, *b* = 22.9321(4) Å, *c* = 25.4294(5) Å, *V*= 11585.3(4) Å^3^, *Z* = 8, *T* = 100(2) K, μ(CuKα) = 0.644 mm^-1^, *Dcalc* = 1.125 g/cm^3^, 356481 reflections measured (6.836° ≤ 2Θ ≤ 161.196°), 12672 unique (*R*_int_ = 0.0562, R_sigma_ = 0.0150) which were used in all calculations. The final *R*_1_ was 0.0725 (>2sigma(I)) and *wR*_2_ was 0.2190 (all data).

**
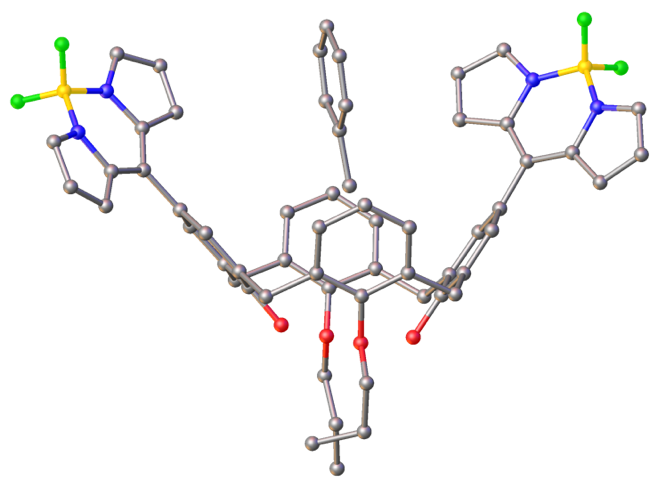
**

**Figure S4.** Single crystal X-ray structure of **2⋅Tol (vertical)** with H atoms omitted for clarity.

**Structure of 2⋅Tol (side-on)**

**Crystal Data for 2⋅Tol (side-on) (CCDC 1962281, *M*=980.68 g/mol):** orthorhombic, space group *P*2_1_2_1_2_1_ (no. 19), *a* = 15.2060(13) Å, *b* = 16.4793(16) Å, *c* = 19.894(2) Å, *V*= 4985.1(8) Å^3^, *Z* = 4, *T* = 150(2) K, μ(Synchrotron), *Dcalc* = 1.307 g/cm^3^, 6061 reflections measured (3.29° ≤ 2Θ ≤ 55.728°), 6061 unique (*R*_int_ = 0.1129, R_sigma_ = 0.0405) which were used in all calculations. The final *R*_1_ was 0.0769 (I > 2σ(I)) and *wR*_2_ was 0.1981 (all data).

**
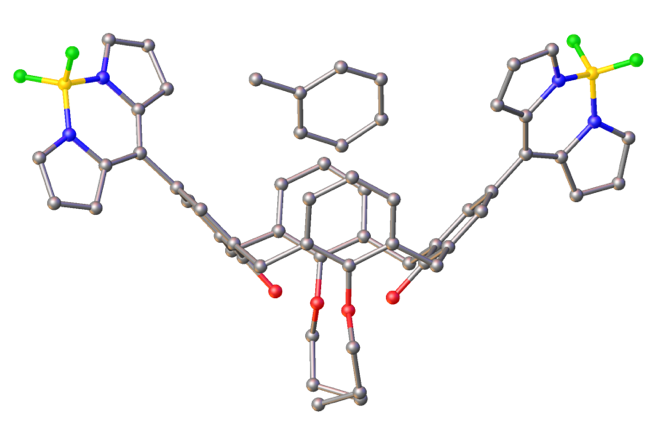
**

**Figure S5.** Single crystal X-ray structure of **2⋅Tol (side-on)** with H atoms omitted for clarity.

**Structure of 4⋅DCM**

**Crystal Data for 4⋅DCM (CCDC 1962282, *M*=1085.68 g/mol):** triclinic, space group *P*-1 (no. 2), *a* = 11.1161(2) Å, *b* = 14.4253(3) Å, *c* = 19.4523(4) Å, *α* = 77.0300(10)°, *β* = 76.1840(10)°, *γ* = 70.8140(10)°, *V*= 2824.29(10) Å^3^, *Z* = 2, *T* = 100(2) K, μ(CuKα) = 1.557 mm^-1^, *Dcalc* = 1.277 g/cm^3^, 64663 reflections measured (4.738° ≤ 2Θ ≤ 149.56°), 11527 unique (*R*_int_ = 0.0836, R_sigma_ = 0.0508) which were used in all calculations. The final *R*_1_ was 0.0736 (I > 2σ(I)) and *wR*_2_ was 0.2020 (all data).

**
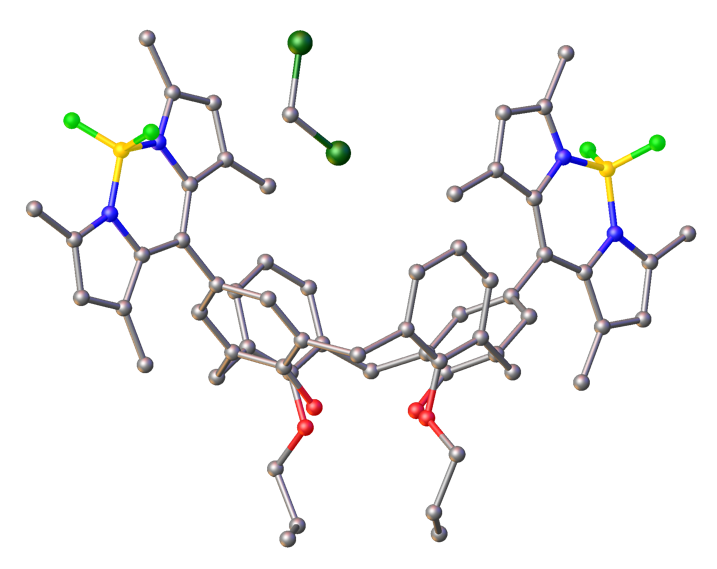
**

**Figure S6.** Single crystal X-ray structure of **4⋅DCM** with H atoms omitted for clarity.

**3. Variable Temperature NMR of 4**


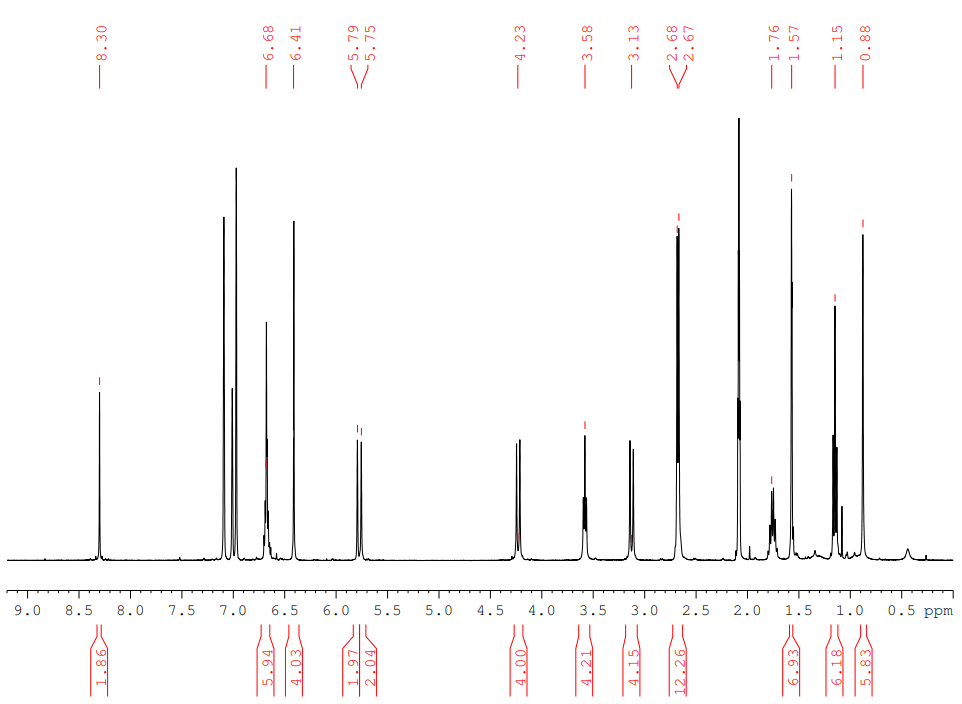
Variable temperature NMR of 4 was performed using a Bruker AVIIIHD 400 MHz NMR spectrometer using toluene-d_8_ as the solvent at both 25 and 60 ^o^C.

**Figure S7.** Full ^1^H NMR of 4 (toluene-d_8_) at 25 ^o^C

**
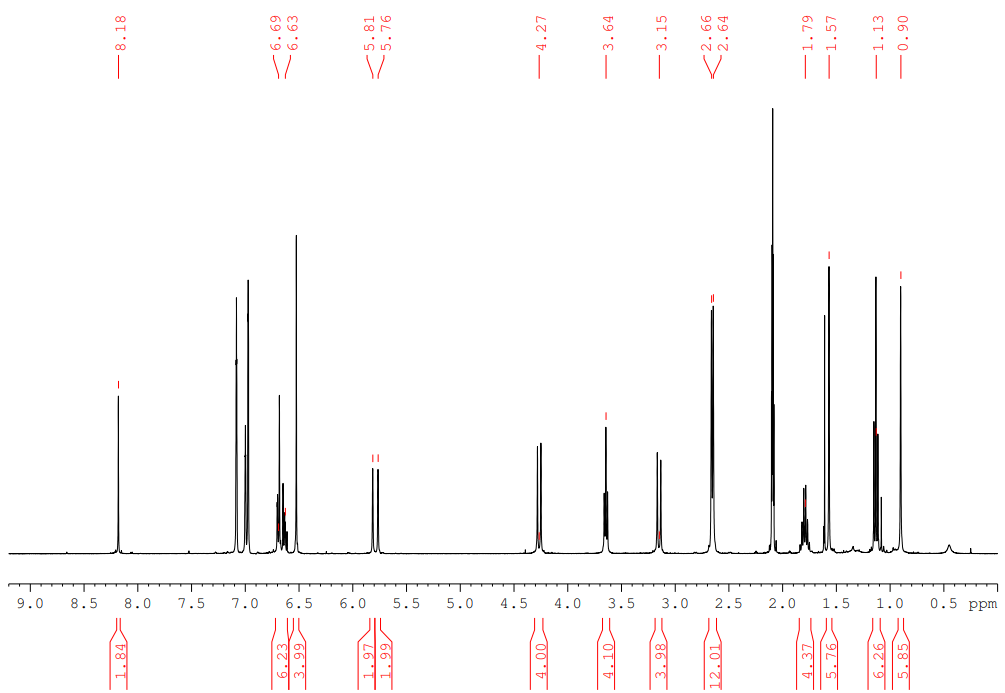
**

**Figure S8.** Full ^1^H NMR of 4 (toluene-d_8_) at 60 ^o^C

**
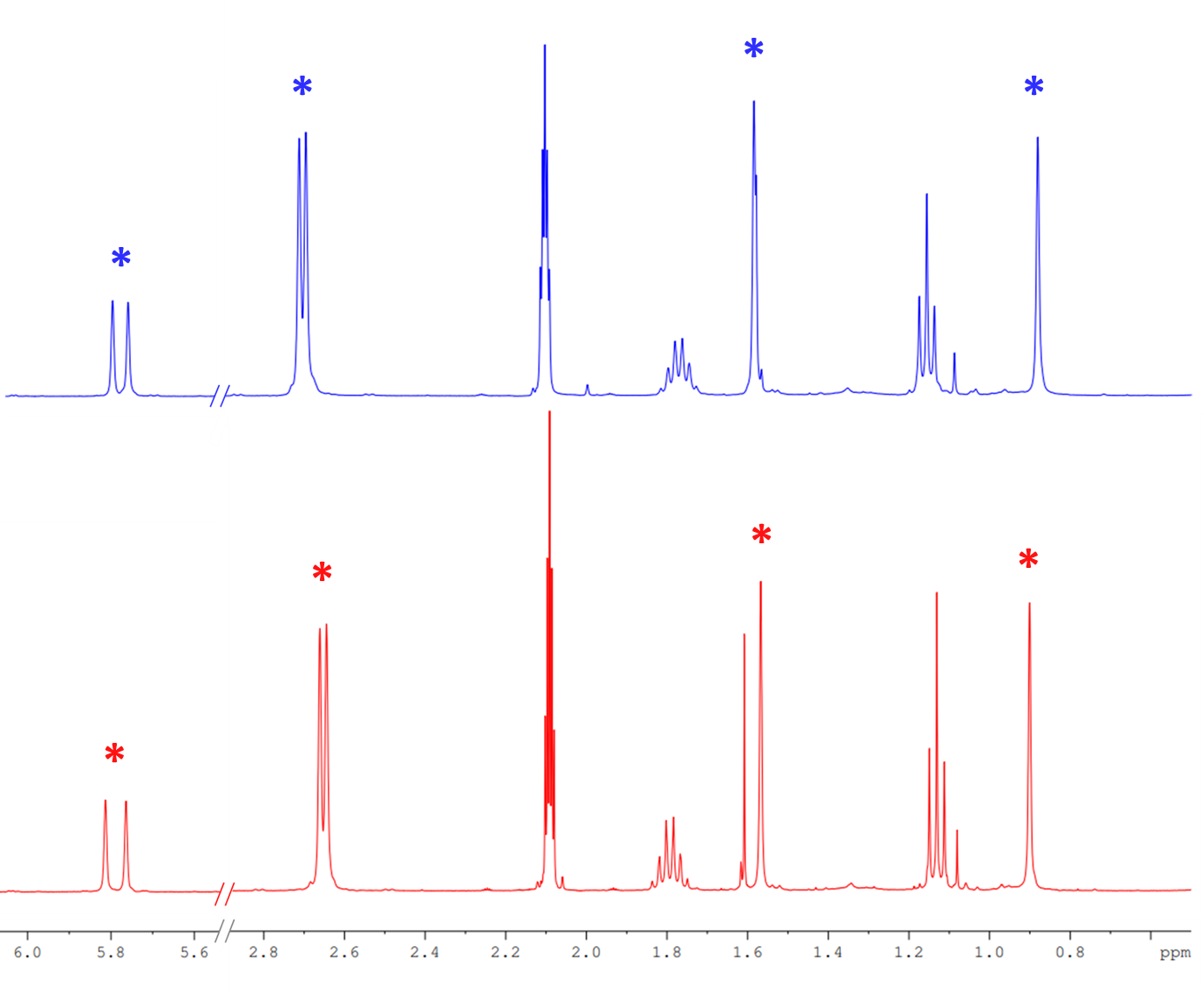
**

**Figure S9.** Truncated ^1^H NMR of 4 (toluene-d_8_) at 25 ^o^C (blue) and 60 ^o^C (red). Peaks split due to self- inclusion of the methyl groups within the cavity are highlighted.

**4. Photophysical Properties of 1 - 5**

**4.1 UV-Vis Absorption Spectra**

UV-Vis absorption spectra were run on a Perkin-Elmer Lambda 25 UV-Vis spectrophotometer using 10mm quartz cuvettes and chloroform as the solvent.


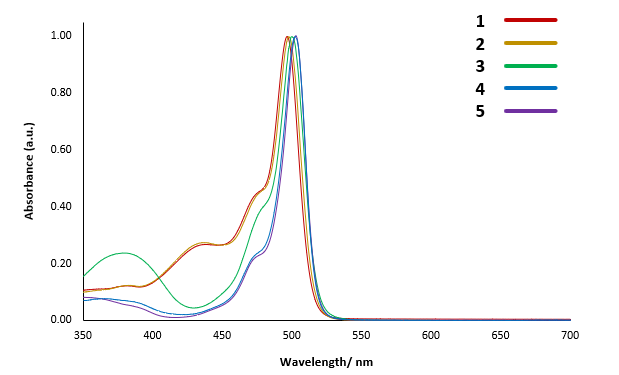


**Figure S10.** Normalised UV-Vis absorption spectra of **1 – 5.**

**4.2 Molar Attenuation Coefficients**

The molar attenuation coefficients, ε_m_ (Table S1), for the BODIPY dyes were determined by preparing solutions of varying dilutions from a parent stock solution and measuring their absorbance at the wavelength of maximum absorbance for each dye. The absorbance and corresponding concentration were then plotted according to the Beer-Lambert-Bouguer law then ε calculated as the gradient of the line of best fit, using the least squares method.

$A= \varepsilon_{m}\mathrm{cl}$

The quoted error is the error associated with the fitting of the line.

**Table S1** Molar attenuation coefficient and associated error for **1 – 5.**

| **Compound** | **ε_m_/ 10^3^ × L mol^-1^ cm^-1^** |
| --- | --- |
| **1** | 55.5 ± 0.7 |
| **2** | 78.0 ± 0.24 |
| **3** | 60.1 ± 0.7 |
| **4** | 131.7 ± 1.1 |
| **5** | 89.0 ± 5.8 |

**4.3 Emission Spectra and PLQY of 1-5**

Emission spectra of **1-5** and their absolute photoluminescence quantum yields (PLQY) were measured using a calibrated Edinburgh Instruments spectrofluorometer (FLS920), equipped with an integrating sphere (Yobin Yvon). The excitation (Excitation grating 300) was achieved with a Xenon lamp (centred at 450 nm) in combination with 2 nm excitation slits. The emission and absorption measurements (Emission grating 750) were recorded with an extended red sensitive detector in cooled housing (PMT, 200-1010 nm) using 15 nm emission slits. Additionally, a 610 nm long pass filter was used in the emission path to record over the 620-850 nm range and avoid second order effects from the monochromator. This was corrected for along with the system response correction for all spectra


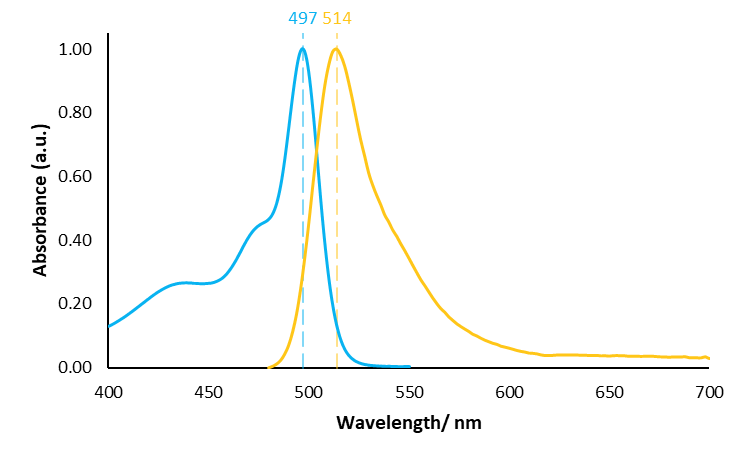


**Figure S11.** Normalised emission spectra of **1** (orange line). Absorption spectra in visible region is shown for comparison (blue line)**.**


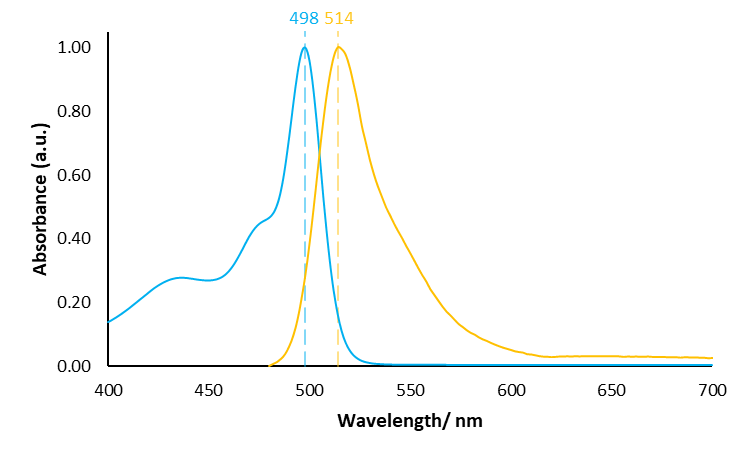


**Figure S12.** Normalised emission spectra of **2** (orange line). Absorption spectra in visible region is shown for comparison (blue line)**.**

**
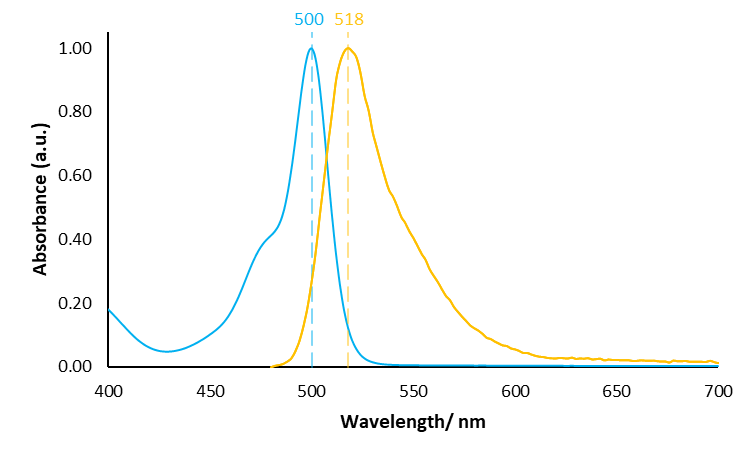
**

**Figure S13.** Normalised emission spectra of **3** (orange line). Absorption spectra in visible region is shown for comparison (blue line)**.**


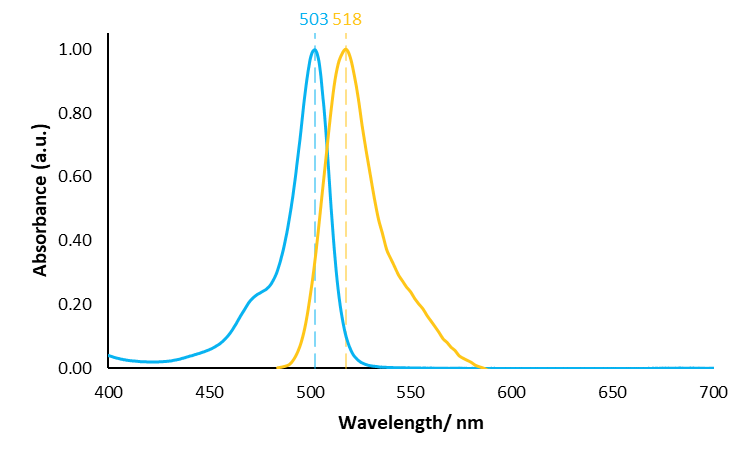


**Figure S14.** Normalised emission spectra of **4** (orange line). Absorption spectra in visible region is shown for comparison (blue line)**.**


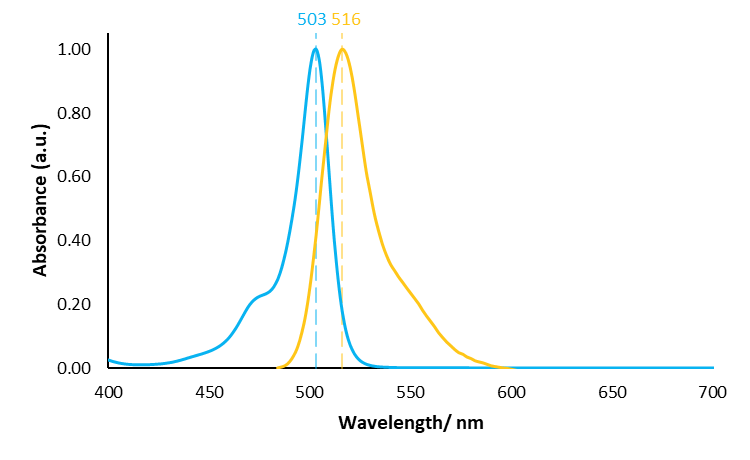


**Figure S15.** Normalised emission spectra of **5** (orange line). Absorption spectra in visible region is shown for comparison (blue line)**.**

The PLQY was measured using the following equation:

$$PLQY=\frac{no. of photons emitted}{no. of photons absorbed}=\frac{L_{sample}}{E_{r}-E_{s}}$$

where L_sample_ is the emission intensity, E_r_ is the intensity of the scattered beam of light not absorbed by the reference (chloroform), E_s_ is the intensity of excitation light not absorbed by the sample. The observed PLQY of **1**-**5** are shown in Table S2.

**Table S2** Experimental results for PLQY determination.

| Compound | Absorbance (%) | Measured PLQY |
| --- | --- | --- |
| **1** | 37.18 | 14.60 |
| **2** | 51.89 | 10.73 |
| **3** | 22.33 | 11.30 |
| **4** | 4.09 | 70.09 |
| **5** | 6.26 | 69.92 |

**5. Computational Studies**

Each of the target molecules was optimised using CAM-B3LYP/6-311G(d,p) starting from the crystal structure coordinates. A polarizable continuum model (PCM) was used to model a chloroform solvent (dielectric constant = 4.7113). Time-dependent (TD) CAM-B3LYP was used to determine the excited singlet electronic states. S_0_, S_1_, and T_1_ states were optimised and the analytical Hessian was confirmed as positive definite to verify the structures as minima. The nature of the excited electronic states was determined using the response eigenvectors with the Kohn-Sham orbitals. All computations were performed using a local version of Gaussian16.^[S10]^

**Table S3** (TD)-CAM-B3LYP/6-311G(d,p) calculated optical properties for target molecules: vertical excitation energies to lowest (bright) state, associated oscillator strengths, adiabatic excitation energies, and (radiative) singlet-triplet energy gaps from lowest triplet T_1_ state.

| **target** | S_1_^vert^ / eV | osc. strength (f) | S_1_^adiab^ / eV | ΔE_S/T_ / kJ mol^-1^ |
| --- | --- | --- | --- | --- |
| **1** | 3.02 | 0.5097 | 2.84 | 148.7 |
| **2** | 3.01 | 0.6086 | 2.84 | 149.1 |
| **3** | 3.00 | 0.5467 | 2.83 | 147.1 |
| **4** | 2.95 | 0.6515 | 2.79 | 146.5 |
| **5** | 2.78 | 0.7567 | 2.68 | 127.7 |


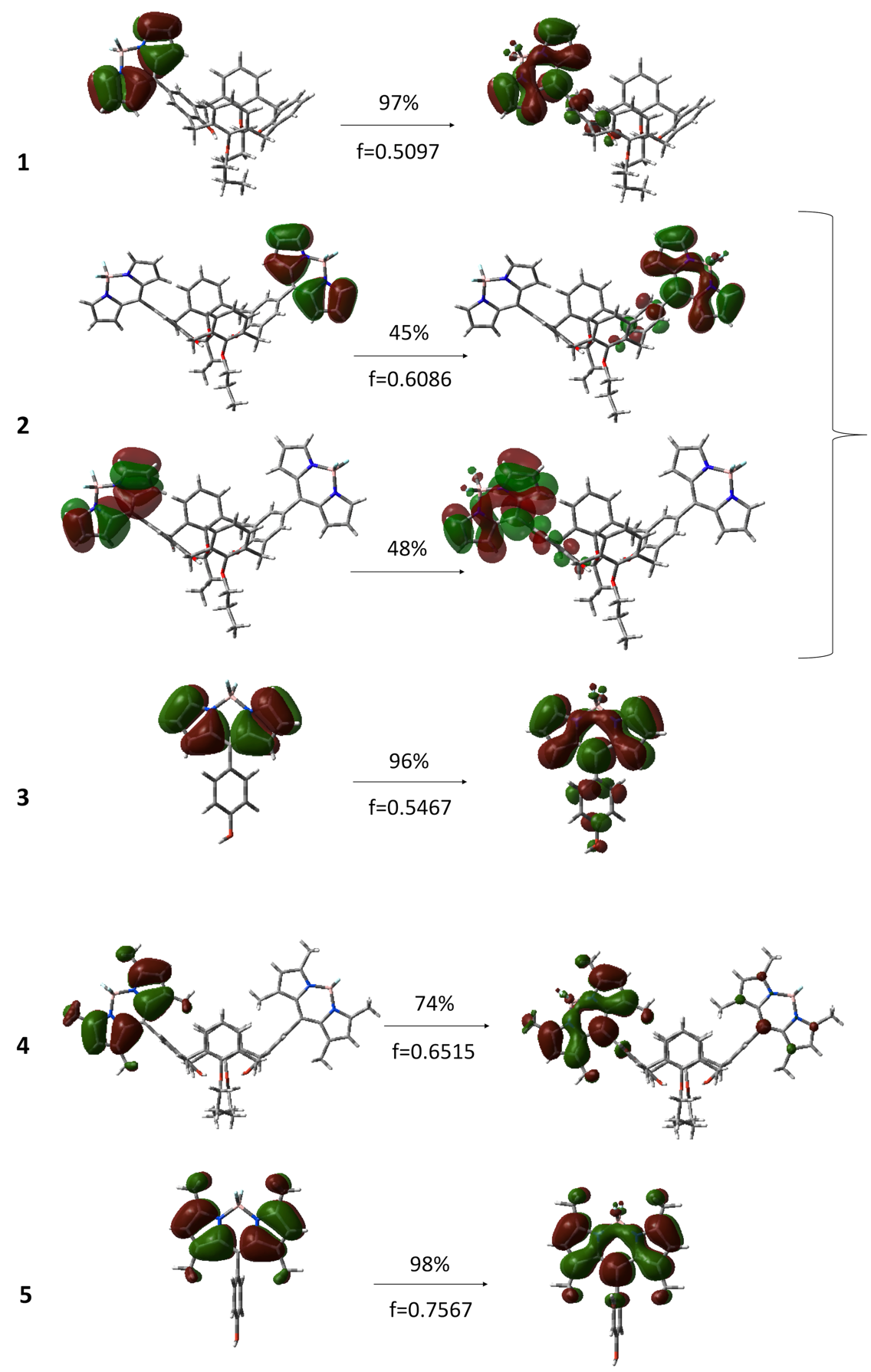


**Figure S16**. Nature of bright S_1_ states of target molecules **1 – 5** obtained from TD-CAM-B3LYP/6-311G(d,p). Dominant particle-hole (p-h) orbitals in Kohn-Sham response eigenvectors are shown together with percentage contribution to transition for each molecule and oscillator strength of the transition. Note that **2** has a pair of equally weighted components locally excited on each BODIPY (with a degenerate partner state with opposite p-h pairs not shown), while **4** has dominant single p-h transition locally excited on a single BODIPY (and a degenerate partner state with dominant p-h local excitation on the other BODIPY, not shown).

**6. Photosensitised ^1^O_2_ Generation and Reaction with 𝛂-Terpinene**

**6.1 Procedures**

**4-BODIPY-Phenol ^1^O_2_ reactions (80 mol% photosensitizer)**

Inside a 25 mL glass vial, 4-BODIPY-Phenol (14.172 mg, 0.05 mmol) was dissolved in chloroform (15 mL). To this, *α*-terpinene (8.51 mg, 0.0625 mmol) was added. The sample was oxygenated under an oxygen stream (20 cm^3^/min) for 5 minutes. An initial sample of solution (150 µL) was taken for NMR examination. The lid of the vial was then tightly sealed. The solution was exposed to a 7.44 W, 500 nm light source (as described previously) at a distance of 60 mm, with a stirring rate of 600 rpm. Samples for NMR analysis (150 µL) were taken at 15 minute intervals until the reaction was observed to be completed. These samples were examined on a Bruker 400 MHz spectrometer using a chloroform suppression sequence.^[S9]^

**5,17-*di*-BODIPY-25,27-*di*-Propoxycalix[4]arene ^1^O_2_ reactions (4.8 mol% photosensitizer)**

Inside a 25 mL glass vial, 5,17-*di*-BODIPY-25,27-*di*-Propoxycalix[4]arene (10.518 mg, 0.012 mmol) was dissolved in chloroform (15 mL). To this, *α*-terpinene (34.1 mg, 0.25 mmol) was added. The sample was oxygenated under an oxygen stream (20 cm^3^/min) for 5 minutes. An initial sample of solution (150 µL) was taken for NMR examination. The lid of the vial was then tightly sealed. The solution was exposed to a 7.44 W, 500 nm light source (as described previously) at a distance of 60 mm, with a stirring rate of 600 rpm. Samples for NMR analysis (150 µL) were taken at 15 minute intervals until the reaction was observed to be completed. These samples were examined on a Bruker 400 MHz spectrometer using a chloroform suppression sequence.

**5-BODIPY-25,27-*di*-Propoxycalix[4]arene ^1^O_2_ reactions (5 mol% photosensitizer)**

Inside a 25 mL glass vial, 5-BODIPY-25,27-*di*-Propoxycalix[4]arene (8.73 mg, 0.0125 mmol) was dissolved in chloroform (15 mL). To this, *α*-terpinene (34.1 mg, 0.25 mmol) was added. The sample was oxygenated under an oxygen stream (20 cm^3^/min) for 5 minutes. An initial sample of solution (150 µL) was taken for NMR examination. The lid of the vial was then tightly sealed. The solution was exposed to a 7.44 W, 500 nm light source (as described previously) at a distance of 60 mm, with a stirring rate of 600 rpm. Samples for NMR analysis (150 µL) were taken at 15 minute intervals until the reaction was observed to be completed. These samples were examined on a Bruker 400 MHz spectrometer using a chloroform suppression sequence.

**5,17-*di*-BODIPY-25,27-*di*-Propoxycalix[4]arene ^1^O_2_ reactions (5 mol% photosensitizer)**

A glass vial was charged with **4** (3.13 mg, 3.125 μmol) and CHCl_3_ (15 mL). To this, *α*-terpinene (8.51 mg, 10 μL, 0.0625 mmol) was added and the sample oxygenated under an oxygen stream of 20 cm^3^/ min for 5 minutes. An initial sample of the solution (150 µL) was withdrawn and diluted with CDCl_3_ for NMR analysis using a CHCl_3_ suppression sequence. The vial was then sealed and irradiated using a 7.44 W 500 – 510 nm light source at a distance of 6 cm, with a constant stirring rate of 600 rpm. Samples for NMR analysis (150 µL) were taken at 2 minute intervals until the reaction was observed to be completed. These samples were examined on a Bruker 400 MHz spectrometer using a chloroform suppression sequence.

**4,4-Difluoro-8-(4-hydroxyphenyl)-1,3,5,7-tetramethyl-4-bora-3a,4adiaza-s-indacene**

**^1^O_2_ reactions (10 mol% photosensitizer)**

A glass vial was charged with photosensitiser (2.13 mg, 6.25 μmol) and CHCl_3_ (15 mL). To this, *α*-terpinene (8.51 mg, 10 μL, 0.0625 mmol) was added and the sample oxygenated under an oxygen stream of 20 cm^3^/ min for 5 minutes. An initial sample of the solution (150 µL) was withdrawn and diluted with CDCl_3_ for NMR analysis using a CHCl_3_ suppression sequence. The vial was then sealed and irradiated using a 7.44 W 500 – 510 nm light source at a distance of 6 cm, with a constant stirring rate of 600 rpm. Samples for NMR analysis (150 µL) were taken at 5 minute intervals until the reaction was observed to be completed. These samples were examined on a Bruker 400 MHz spectrometer using a chloroform suppression sequence.


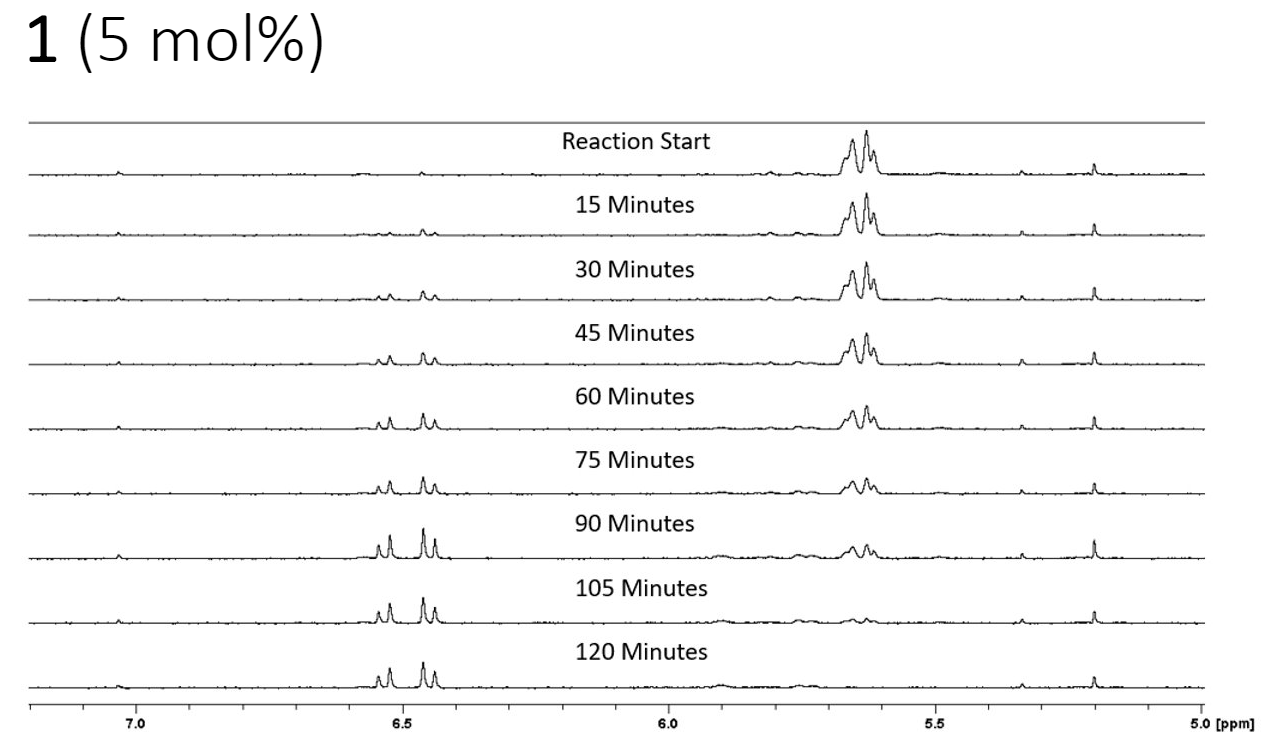


**Figure S17.** Stacked NMR plots showing consumption of *α*-terpinene over the course of approximately 2 hours in the presence of **1** as a photosensitizing host catalyst (at 5 mol%).

**6.2 Overnight Bleaching Tests**

Solutions of chloroform (15 mL) and compounds (**1** – **3**) were prepared as described in the above experimental information. These solutions were irradiated with 500 nm light using the previously described conditions for a period of 15 hours. After this time, an appropriate amount of *α*-terpinene was added to the solution and irradiation continued. The reaction was monitored at 15 minute intervals using ^1^H NMR. Figures S8 – S10 show that there is little change to photosensitizing / H:G photocatalytic behaviour due to photobleaching.

**Figure S18.** Photocatalytic runs using **1** (5 mol%) as freshly synthesised (solid diamonds) and after overnight photobleaching (hollow diamonds).

**Figure S19.** Photocatalytic runs using **2** (4.8 mol%) as freshly synthesised (solid triangles) and after overnight photobleaching (hollow triangles).

**Figure S20.** Photocatalytic runs using **3** (80 mol%) as freshly synthesised (solid spheres) and after overnight photobleaching (hollow spheres).

**6.3 Control Experiments**

**Experiment to show the dependency of light on the catalytic process**

A solution of chloroform (15 mL) containing *α*-terpinene (34.1 mg, 0.25 mmol) and a 5 mol% loading of **1** (8.73 mg, 0.0125 mmol) was prepared as described previously. This solution was irradiated with 500 nm light for 30 minutes using the previously described conditions, with samples being analysed by NMR at 15-minute intervals. After this time, the light source was switched off and further samples were taken at 90 minutes and 170 minutes, After this time, the light was switched back on and samples for analysis were again collected at 15 minute intervals. 200 minutes after the start of the reaction, the light was switched off and the mixture left to stir for a further 1000 minutes. After this time, an NMR sample was taken and the light turned on once more. Samples were then taken every 15 minutes until the reaction was observed to be completed. From this experiment we can clearly see dependency of the catalyst on light.

**Experiment to monitor the temperature of the catalytic reaction setup**

A solution of chloroform (15 mL) containing *α*-terpinene (34.1 mg, 0.25 mmol) and a 5 mol% loading of **1** (8.73 mg, 0.0125 mmol) was prepared as described previously. This solution was irradiated with 500 nm light using the previously described conditions. Every 15 minutes, before an NMR sample was taken, the temperature of the reaction vial was monitored with a PowerTec Energy Infrarot Thermometer. NMR samples were then taken as previously described and the reaction continued. As can be seen from Figure S11, the temperature rises by around 8 °C over the course of approximately 2 hours using the methods we have employed here. We believe this is not a significant factor in the activity of the host photosensitizers used in this study, but in future work we will design and use a temperature-controlled housing system (using a fan / ventilation) to ensure that this remains constant throughout.

**Figure S21.** Change in reaction temperature over the course of a photocatalytic run performed with **1** (5 mol%).

**Experiment to monitor the dependency of a photosensitizing catalyst on the conversion of *α*-terpinene to ascaridole**

A solution of chloroform (15 mL) containing *α*-terpinene (34.1 mg, 0.25 mmol) was prepared. This solution was irradiated with 500 nm light using the previously described conditions for a period of 2 hours. After this time, a 5 mol% loading of **1** (8.73 mg, 0.0125 mmol) was added to the solution and the irradiation continued. Monitoring of this experiment showed no reaction progress in an absence of catalyst, and standard completion rates once the catalyst had been added.

**Competitive host experiments**

**1:1 ratio of 1:DiPrC[4]**

Inside a 25 mL glass vial, 5-BODIPY-25,27-*di*-propoxycalix[4]arene (8.73 mg, 0.0125 mmol) and *di*‑propoxycalix[4]arene (6.358mg, 0.0125 mmol) were combined in a vial and dissolved with chloroform (15 mL). To this, *α*-terpinene (34.1 mg, 0.25 mmol) was added. The sample was oxygenated under an oxygen stream (20 cm^3^/min) for 5 minutes. An initial sample of solution (150 µL) was taken for NMR examination. The lid of the vial was then tightly sealed. The solution was exposed to a 7.44 W, 500 nm light source (as described previously) at a distance of 60 mm, with a stirring rate of 600 rpm. Samples for NMR analysis (150 µL) were taken at 15 minute intervals until the reaction was observed to be completed. These samples were examined on a Bruker 400 MHz spectrometer using a chloroform suppression sequence.

**1:10 ratio of 1:DiPrC[4]**

Inside a 25 mL glass vial, 5-BODIPY-25,27-*di*-propoxycalix[4]arene (8.73 mg, 0.0125 mmol) and *di*‑propoxycalix[4]arene (63.583 mg, 0.125 mmol) were combined in a vial and dissolved with chloroform (15 mL). To this, *α*-terpinene (34.1 mg, 0.25 mmol) was added. The sample was oxygenated under an oxygen stream (20 cm^3^/min) for 5 minutes. An initial sample of solution (150 µL) was taken for NMR examination. The lid of the vial was then tightly sealed. The solution was exposed to a 7.44 W, 500 nm light source (as described previously) at a distance of 60 mm, with a stirring rate of 600 rpm. Samples for NMR analysis (150 µL) were taken at 15 minute intervals until the reaction was observed to be completed. These samples were examined on a Bruker 400 MHz spectrometer using a chloroform suppression sequence.

**Figure S22.** Plot of *α*-terpinene consumption as a function of time in the presence of photosensitiser **1** at 5 mol% with varying mol% ratios of DiPrC[4] (inset).

**1:10 ratio of *α*-terpinene:*p*-cymene**

Inside a 25 mL glass vial, 5-BODIPY-25,27-*di*-propoxycalix[4]arene (8.73 mg, 0.0125 mmol) and *p*‑cymene (335.6 mg, 2.5 mmol) were combined in a vial and the total volume of solution was made up to 15 mL with chloroform. To this, *α*-terpinene (34.1 mg, 0.25 mmol) was added. The sample was oxygenated under an oxygen stream (20 cm^3^/min) for 5 minutes. An initial sample of solution (150 µL) was taken for NMR examination. The lid of the vial was then tightly sealed. The solution was exposed to a 7.44 W, 500 nm light source (as described previously) at a distance of 60 mm, with a stirring rate of 600 rpm. Samples for NMR analysis (150 µL) were taken at 15 minute intervals until the reaction was observed to be completed. These samples were examined on a Bruker 400 MHz spectrometer using a chloroform suppression sequence.

**1:25 ratio of *α*-terpinene:*p*-cymene**

Inside a 25 mL glass vial, 5-BODIPY-25,27-*di*-propoxycalix[4]arene (8.73 mg, 0.0125 mmol) and *p*‑cymene (838.9 mg, 6.25 mmol) were combined in a vial and the total volume of solution was made up to 15 mL with chloroform. To this, *α*-terpinene (34.1 mg, 0.25 mmol) was added. The sample was oxygenated under an oxygen stream (20 cm^3^/min) for 5 minutes. An initial sample of solution (150 µL) was taken for NMR examination. The lid of the vial was then tightly sealed. The solution was exposed to a 7.44 W, 500 nm light source (as described previously) at a distance of 60 mm, with a stirring rate of 600 rpm. Samples for NMR analysis (150 µL) were taken at 15 minute intervals until the reaction was observed to be completed. These samples were examined on a Bruker 400 MHz spectrometer using a chloroform suppression sequence.

**Figure S23.** Plot of α-terpinene consumption as a function of time in the presence of photosensitiser **1** at 5 mol% with varying ratios of *p*-cymene:*α*-terpinene (inset).

In the CHCl_3_ suppression experiments with competitive guest molecule *p*-cymene present (above), the aromatic proton peak for *p*-cymene is supressed as it falls in a region sufficiently similar to chloroform, and the alkyl peaks associated with the compound are outside the spectral window of the experiment. However this leads to a large amount of spectral folding with sign inversion in the upfield portion of the spectra resulting in non-linearity in the baseline. As such, the data has been manually baseline corrected for the purpose of these experiments but leads to an unknown amount of integral error.


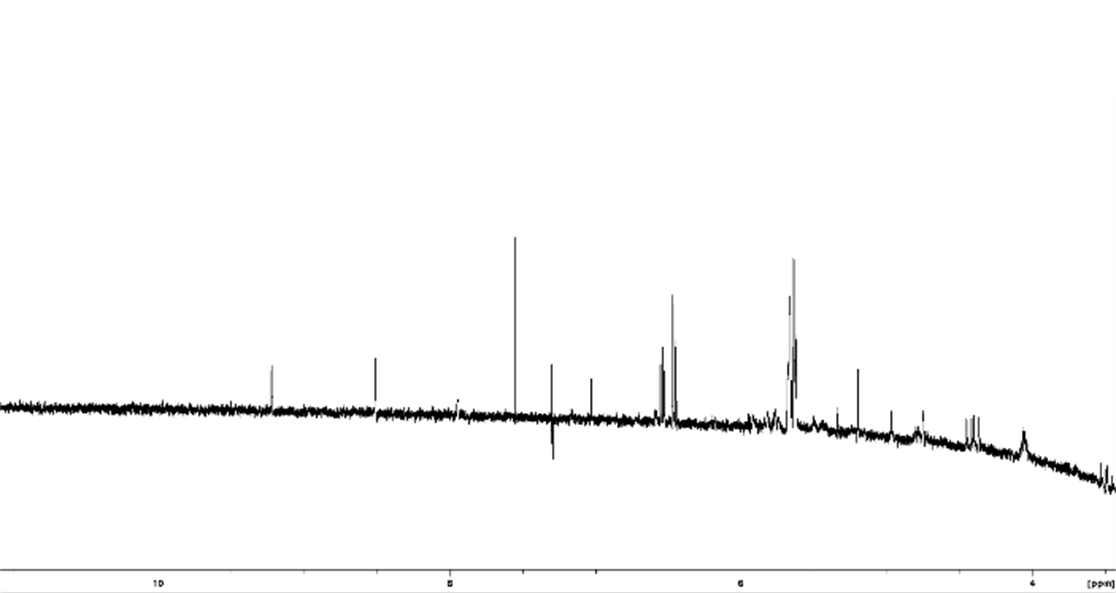
 **Figure S24.** ^1^H NMR of **1** (5 mol%) and *p*-cymene (25 eq relative to *α*-terpinene) at T = 60 mins with no manual baseline correction.


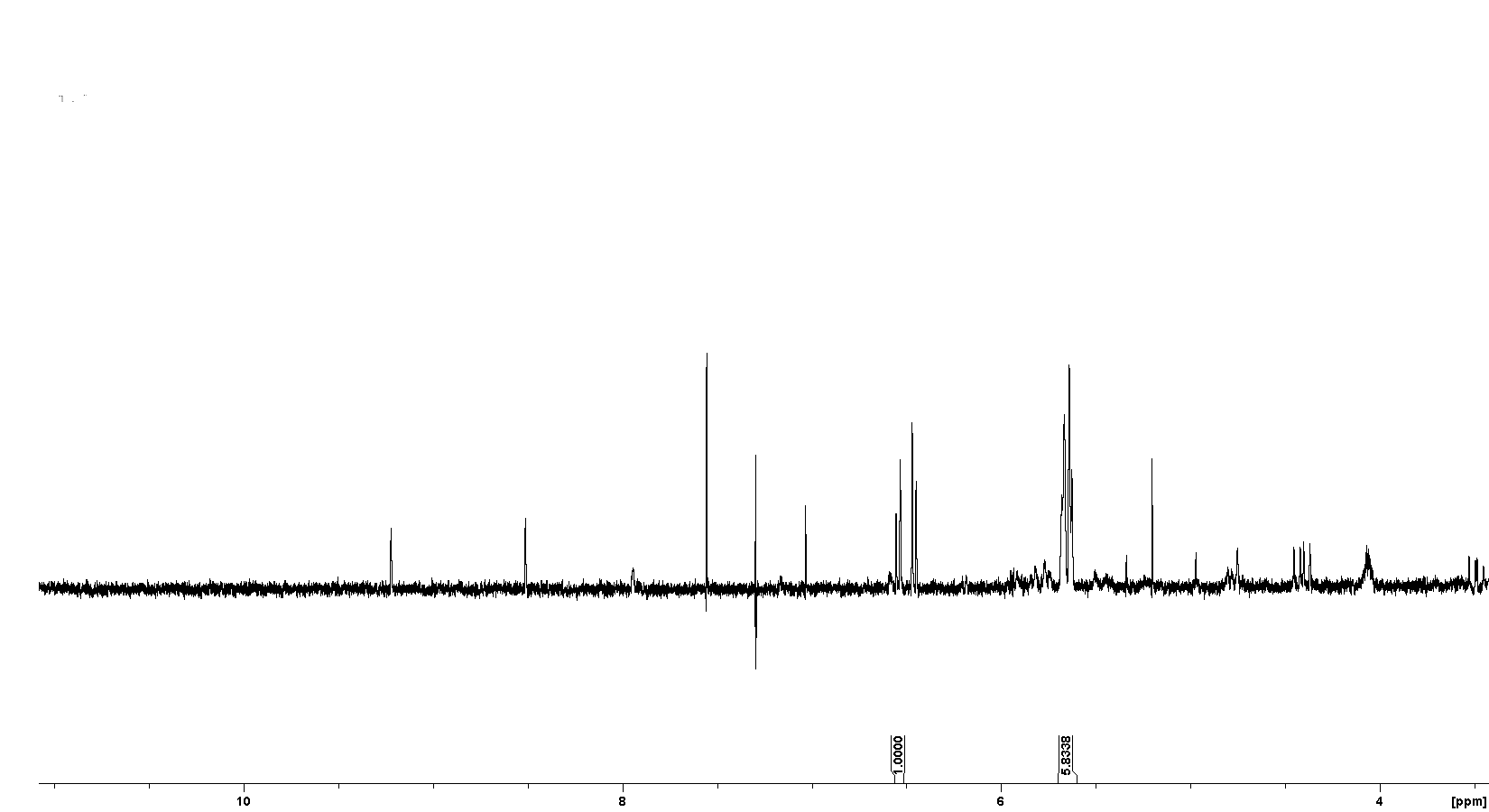


**Figure S25.** ^1^H NMR of **1** (5 mol%) and *p*-cymene (25 eq relative to *α*-terpinene) at T = 60 mins with manual baseline correction.

***α*-Terpinene consumption in the presence of 3 (80 mol%) and DiPrC[4] (40 mol%)**

Inside a 25 mL glass vial, 4-BODIPY-phenol (14.172 mg, 0.05 mmol) and *di*-propoxycalix[4]arene (12.72 mg, 0.025 mmol) were combined and dissolved in chloroform (15 mL). To this, *α*-terpinene (8.51 mg, 0.0625 mmol) was added. The sample was oxygenated under an oxygen stream (20 cm^3^/min) for 5 minutes. An initial sample of solution (150 µL) was taken for NMR examination. The lid of the vial was then tightly sealed. The solution was exposed to a 7.44 W, 500 nm light source (as described previously) at a distance of 60 mm, with a stirring rate of 600 rpm. Samples for NMR analysis (150 µL) were taken at 15 minute intervals until the reaction was observed to be completed. These samples were examined on a Bruker 400 MHz spectrometer using a chloroform suppression sequence.

**Figure S26.** A comparative plot of α-terpinene consumption as a function of time in the presence of photosensitiser **3** at 80 mol% both in the presence and absence of DiPrC[4] (inset).

**7. Binding studies via Stern-Volmer fluorescence quenching**

**Figure S27.** Quenching curves (top) and Stern-Volmer plots (bottom) of **1** versus *α*-terpinene (0, 2, 4, 6, 8, 10, 20 eq.).

**Figure S28.** Quenching curves (top) and Stern-Volmer plots (bottom) of **2** versus α-terpinene (0, 2, 4, 6, 8, 10, 20 eq.).

**Figure S29.** Quenching curves (top) and Stern-Volmer plots (bottom) of **4** versus α-terpinene (0, 2, 4, 6, 8, 10, 20 eq.).

**Figure S30.** Quenching curves (top) and Stern-Volmer plots (bottom) of **5** versus α-terpinene (0, 2, 4, 6, 8, 10, 20 eq.).

The fluorescence measurements were performed on a fluorimeter with high sensitivity which required dilution of the photoactive moiety to accommodate the intensity maxima limit of the instrument. Hence, while the concentrations of the photoactive species reported in Figures S26-S29 differ from each other, the stepwise addition of 2 incremental equivalents was performed using *ad hoc* stock solutions of *α-*terpinene, to maintain stoichiometric ratios exact while minimising volume change in the cuvette (which was still accounted for in the total volume of solution).

**8. Spectroscopic Characterisation of Synthesised Products**

**NMR Spectra for compound 1**

**
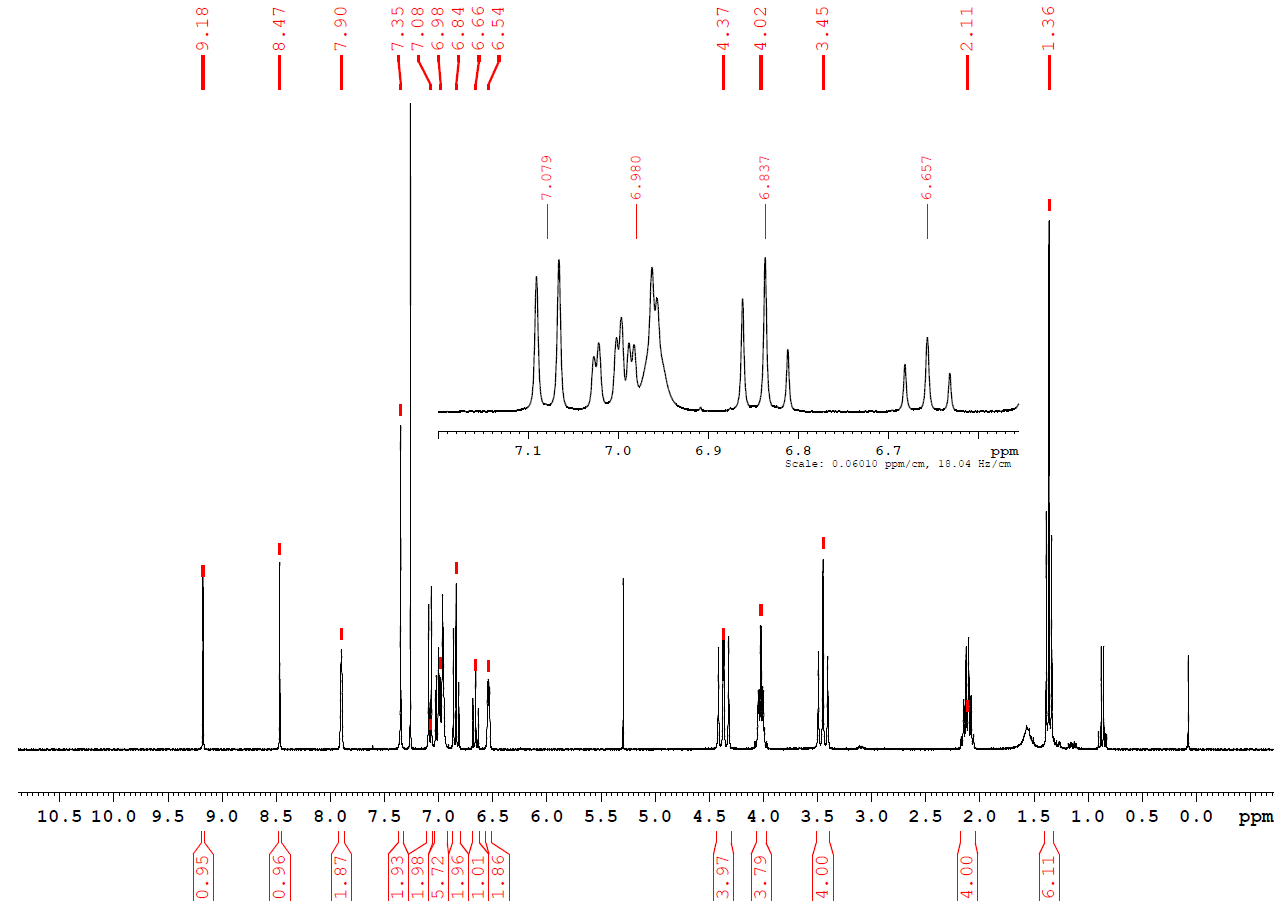
**

**NMR Spectra for compound 2**


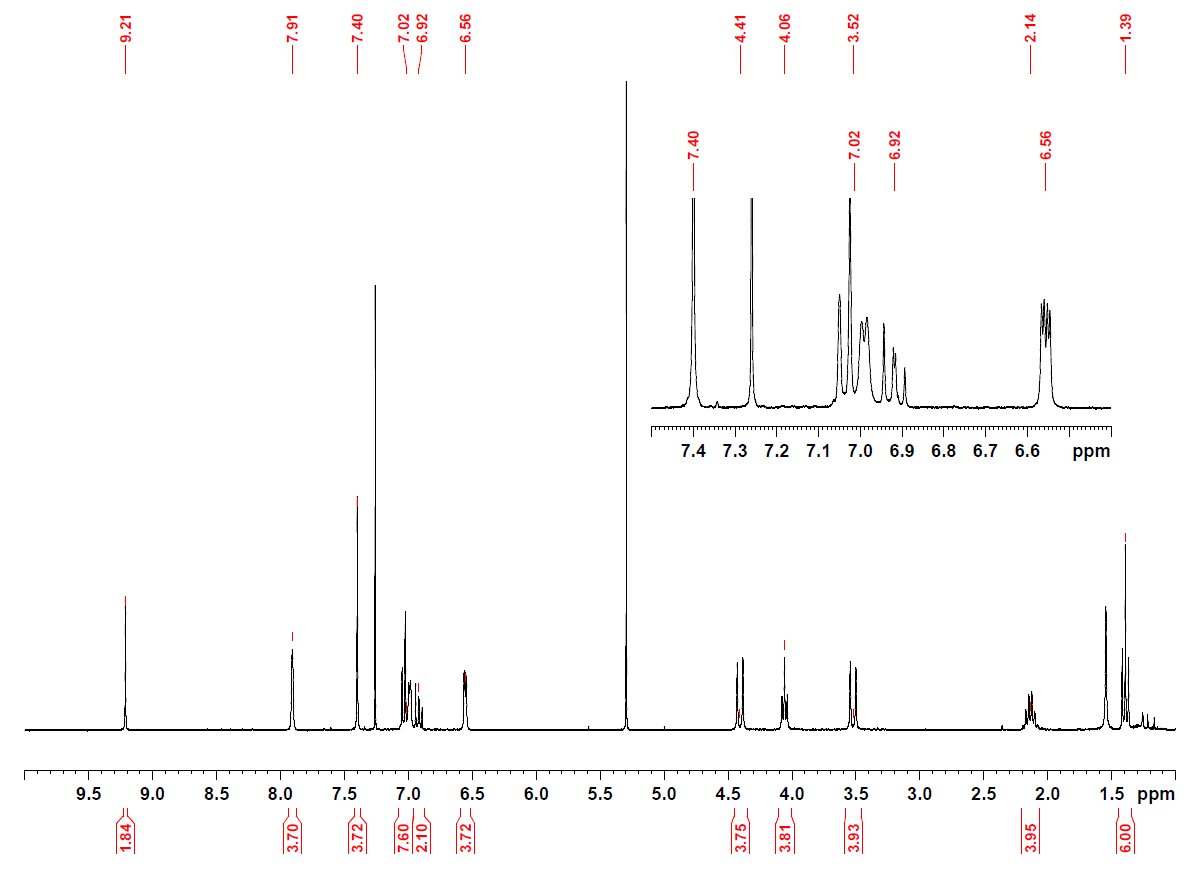


**NMR Spectra for compound 3**


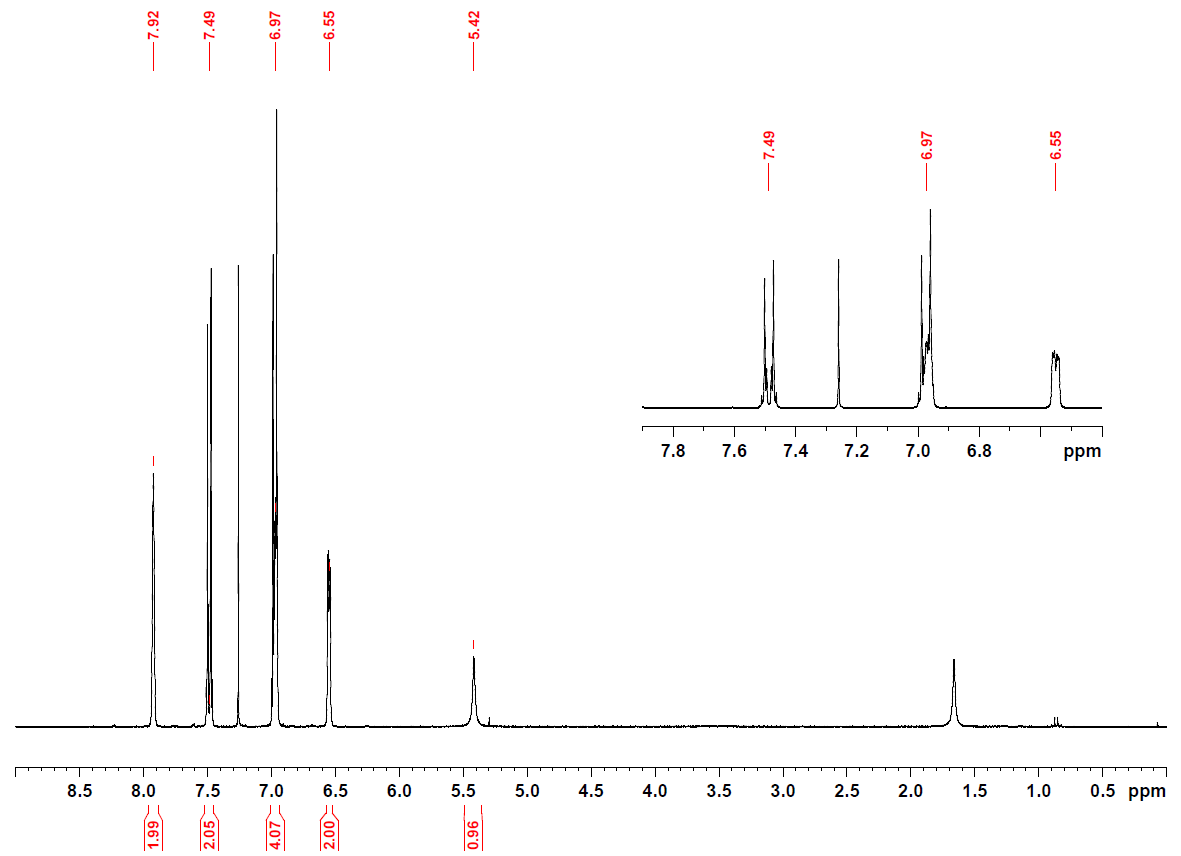


**NMR Spectra for Compound 4**


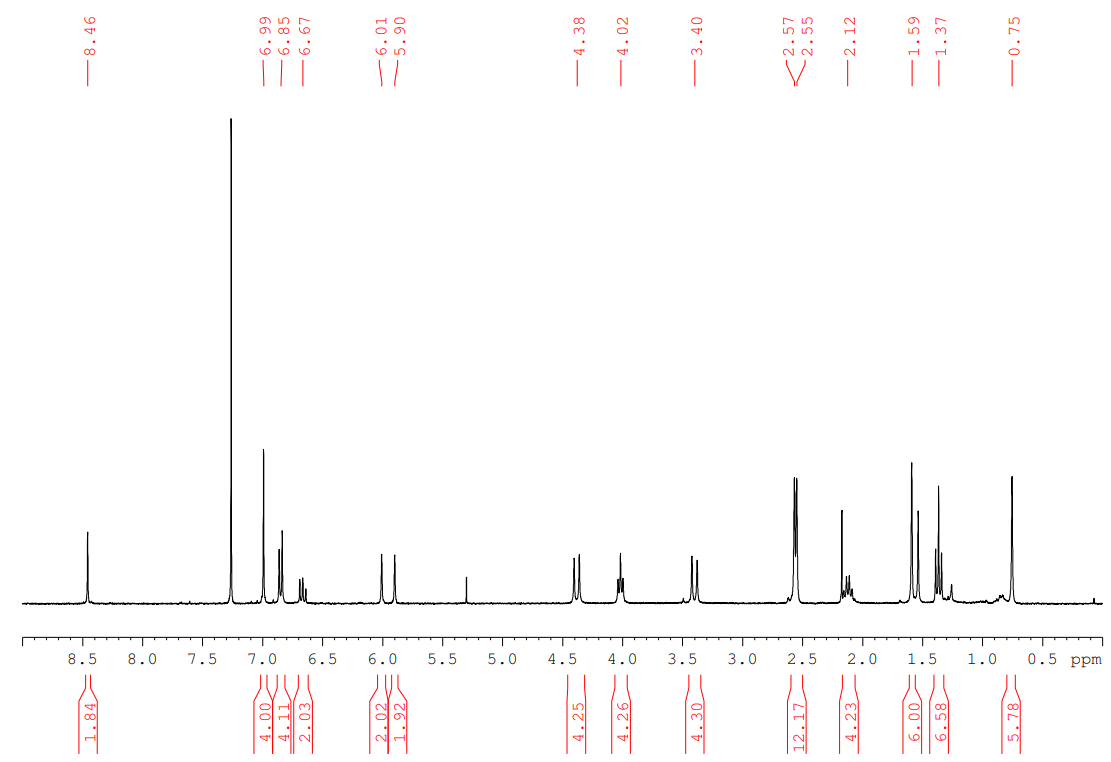


**NMR Spectra for Compound 5**


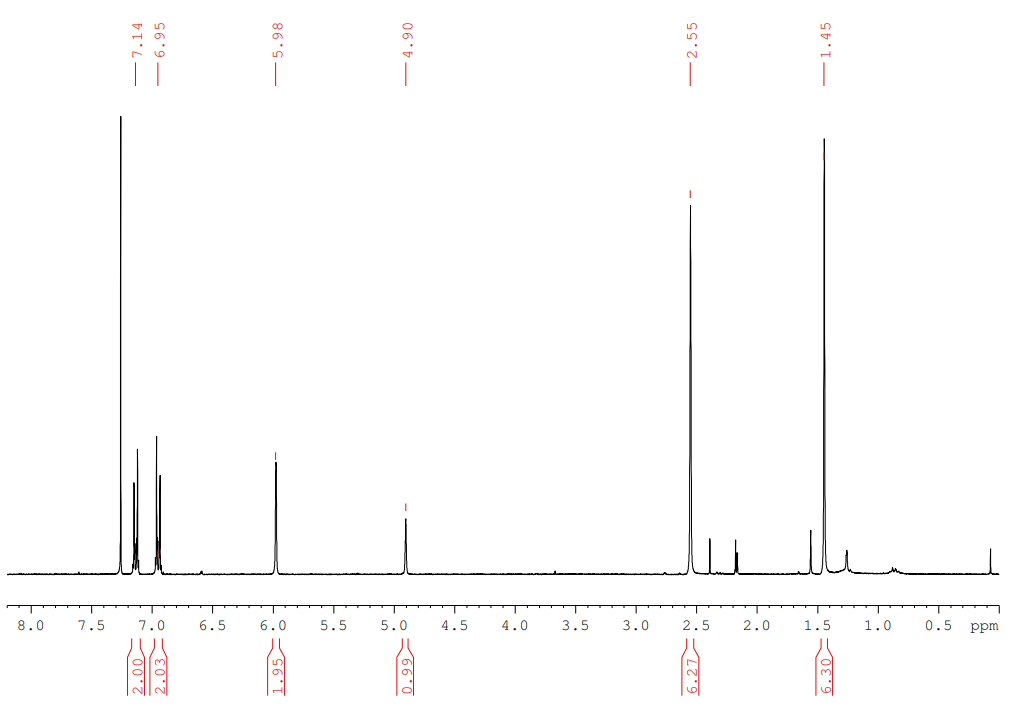


**9. References**

[S1] T. Nishioka, T. Arimura, Y. Suga, S. Murata, M. Tachiya, M. Goto, *Bull. Chen. Soc. Jpn.* **2001**, *74*, 2435-2436.

[S2] C. N. Baki, E. U. Akkaya, *J. Org. Chem.* **2001**, *66*, 1512-1513.

[S3] N. R. Cha, S. Y. Moon, S.-K. Chang, *Tet. Lett.* **2003**, *44*, 8265-8268.

[S4] H. J. Kim, J. S. Kim, *Tet. Lett.* **2006**, *47*, 8051-8055.

[S5] J.-D. van Loon, A. Arduini, L. Coppi, W. Verboom, A. Pochini, R. Ungaro, S. Harkema and D. N. Reinhoudt, *J. Org. Chem*, **1990**, *55*, 5639–5646.

[S6] H. M. Chawla, S. Kumar, N. Pant, A. Santra, K. Srinivas, N. Kumar, D. S. Black, *J. Incl. Phenom. Macrocycl. Chem.* **2011**, *71*, 169-178.

[S7] M. Baruah, W. Qin, N. Basarić, W. M. De Borggraeve, N. Boens, *J. Org. Chem.* **2005**, *70*, 4152-4157.

[S8] D. Prasannan, D. Raghav, S. Sujatha, H. H. Kumar, K. Rathinasamy, C. Arunkumar, *RSC Adv.* **2016**, *6*, 80808-80824.

[S9] J. M. Tobin, J. Liu, H. Hayes, M. Demleitner, D. Ellis, V. Arrighi, Z. Xu and F. Vilela, *Polym. Chem.*, **2016**, *7*, 6662-6670.

[S10] Gaussian 16, Revision A.03, M. J. Frisch, G. W. Trucks, H. B. Schlegel, G. E. Scuseria, M. A. Robb, J. R. Cheeseman, G. Scalmani, V. Barone, G. A. Petersson, H. Nakatsuji, X. Li, M. Caricato, A. V. Marenich, J. Bloino, B. G. Janesko, R. Gomperts, B. Mennucci, H. P. Hratchian, J. V. Ortiz, A. F. Izmaylov, J. L. Sonnenberg, D. Williams-Young, F. Ding, F. Lipparini, F. Egidi, J. Goings, B. Peng, A. Petrone, T. Henderson, D. Ranasinghe, V. G. Zakrzewski, J. Gao, N. Rega, G. Zheng, W. Liang, M. Hada, M. Ehara, K. Toyota, R. Fukuda, J. Hasegawa, M. Ishida, T. Nakajima, Y. Honda, O. Kitao, H. Nakai, T. Vreven, K. Throssell, J. A. Montgomery, Jr., J. E. Peralta, F. Ogliaro, M. J. Bearpark, J. J. Heyd, E. N. Brothers, K. N. Kudin, V. N. Staroverov, T. A. Keith, R. Kobayashi, J. Normand, K. Raghavachari, A. P. Rendell, J. C. Burant, S. S. Iyengar, J. Tomasi, M. Cossi, J. M. Millam, M. Klene, C. Adamo, R. Cammi, J. W. Ochterski, R. L. Martin, K. Morokuma, O. Farkas, J. B. Foresman, and D. J. Fox, Gaussian, Inc., Wallingford CT, **2016**.
